# Uncovering the hidden world of riverbed sediments: The role of sediment heterogeneity and cross-bar channel fills in the hydrogeochemical dynamics of the hyporheic zone

**DOI:** 10.1016/j.jhydrol.2024.132062

**Published:** 2024-11

**Authors:** Jeffery T. McGarr, Pei Li, Robert G. Ford, Teagan Kleman, Colton Fields, Julie Hobbs, Lydia Lupton, Emma Poston, Thomas Marsh, Leah Trutschel, Ken M. Fritz, Annette Rowe, Corey D. Wallace, Dylan Ward, Daniel M. Sturmer, Craig Dietsch, Margaret Naber, Bob K. Lien, Mohamad Reza Soltanian

**Affiliations:** a Department of Geosciences, University of Cincinnati, Cincinnati, OH, USA; b Office of Research and Development, U.S. Environmental Protection Agency, Cincinnati, OH, USA; c Department of Biological Sciences, University of Cincinnati, Cincinnati, OH, USA; d RSI EnTech, Grand Junction, CO, USA; e Department of Environmental Engineering, University of Cincinnati, Cincinnati, OH, USA

**Keywords:** Hyporheic exchange, Solute mixing, Geochemical hotspots, Riverbed, Geophysical imaging

## Abstract

Groundwater-surface water interaction (hyporheic exchange) is critical in numerous hydrogeochemical processes; however, hyporheic exchange is difficult to characterize due to the various spatial (e.g., sedimentary architecture) and temporal (e.g., stage fluctuations) variables that influence it. This interdisciplinary study brings forth novel insights by integrating various methodologies including geophysical surveys, physical and chemical sediment characterization, and water chemistry analysis to explore the interplay of the numerous facets governing hyporheic zone processes within a compound bar deposit. The findings reveal distinct sedimentary facies and geochemical zones within the compound bar, driven by the sedimentary architecture. Cross-bar channel fills are identified as critical structures influencing hydrogeochemical dynamics, acting as baffles to groundwater flow and modulating nutrient transformations. Geophysical imaging and hydrogeochemical analyses highlight the complex interplay between sediment characteristics and subsurface hydraulic connectivity, emphasizing the role of sediment heterogeneity in controlling hyporheic exchange and solute mixing. The study concludes that sediment heterogeneity, particularly the presence of cross-bar channel fills, plays a pivotal role in the hydrogeochemical dynamics of the hyporheic zone. These structures significantly influence hyporheic flow paths, solute residence times, and nutrient cycling, underscoring the necessity to consider the fine-scale sedimentary architecture in models of hyporheic exchange. The findings contribute to a deeper understanding of riverine ecosystem processes, offering insights that can inform management strategies for water quality and ecological integrity.

## 1. Introduction

The hyporheic zone serves as a critical interface between river water and groundwater that is fundamental to the ecology of riverine systems (Krause et al., 2022; Boano et al., 2014; Cardenas, 2015; Graham et al., 2018; Stegen et al., 2018). The hyporheic zone encompasses a complex network of flow paths that vary widely in scale, from millimeters to hundreds of meters, and in residence time, from seconds to years (Harvey and Bencala, 1993; Thibodeaux and Boyle, 1987; Harvey and Wagner, 2000). These flow paths are dictated by pressure gradients that are driven by a variety of spatial and temporal controls, including bed form topography and geometry, channel morphology, sediment heterogeneity (e.g., hydraulic conductivity, *K* ), and fluctuations in river stage and discharge (Zhou et al., 2014; Stonedahl et al., 2010; Johnson et al., 2015; Singh et al., 2019; Cardenas et al., 2004; Angermann et al., 2012; Lewandowski et al., 2011a). Physical and chemical sediment heterogeneity within the hyporheic zone can influence the fate and transport of nutrients and contaminants as well as microbial community structures (Groffman et al., 2005; Harvey et al., 2012; Marzadri et al., 2012; Trauth et al., 2015; Riml et al., 2013; Lewandowski and Nützmann, 2010). Previous work unveiled the hyporheic zone’s paramount importance, particularly concerning its intricate hydrogeochemical dynamics (Zhao et al., 2021; Arora et al., 2022; Nelson et al., 2019; Saup et al., 2019; Singh et al., 2019; Lewandowski et al., 2011b). Despite its significance, characterizing exchange and solute fate and transport within the hyporheic zone is a challenge (Menichino, 2023; Drouin et al., 2021). A key driver of hydrogeochemical processes in the hyporheic zone is the sedimentary architecture and the associated underlying physical and chemical heterogeneity (Pryshlak et al., 2015; Sawyer and Cardenas, 2009; Zhou et al., 2014).

In the exploration of riverine environments, an enhanced understanding of the controlling influence of sedimentary structures on hydrogeochemical processes is essential. While the relationship between channel morphology and hyporheic exchange cannot be ignored (Cardenas et al., 2004; Hester and Doyle, 2008; Stonedahl et al., 2010), the underlying sedimentary heterogeneity can have a larger impact on hyporheic flow (Zhou et al., 2014). Previous investigations of riparian floodplain sedimentary heterogeneity found that preferential flow pathways adjacent to low-permeability facies (i.e., bodies of sediments Soltanian and Ritzi, 2014) corresponded with distinct oxidation–reduction (redox) zonation indicative of enhanced biogeochemical activity (i.e., hotspots) (Wallace et al., 2021; Wallace and Soltanian, 2021b). Preferential flow paths dictated by highly permeable facies types and their connectivity can also control solute residence times (Sawyer and Cardenas, 2009; Pryshlak et al., 2015). Accurate characterization of sedimentary heterogeneity is critical, as incorporating observational data into hyporheic flow models can alter solute residence times by orders of magnitude (Ward et al., 2013). It is also important to identify which scales of sedimentary heterogeneity are most important to capture hyporheic flux and solute residence times. Despite the previous work on hyporheic dynamics, partially-inundated, large-scale compound bar deposits have not yet received significant attention in the context of hyporheic exchange.

**Fig. 1.**
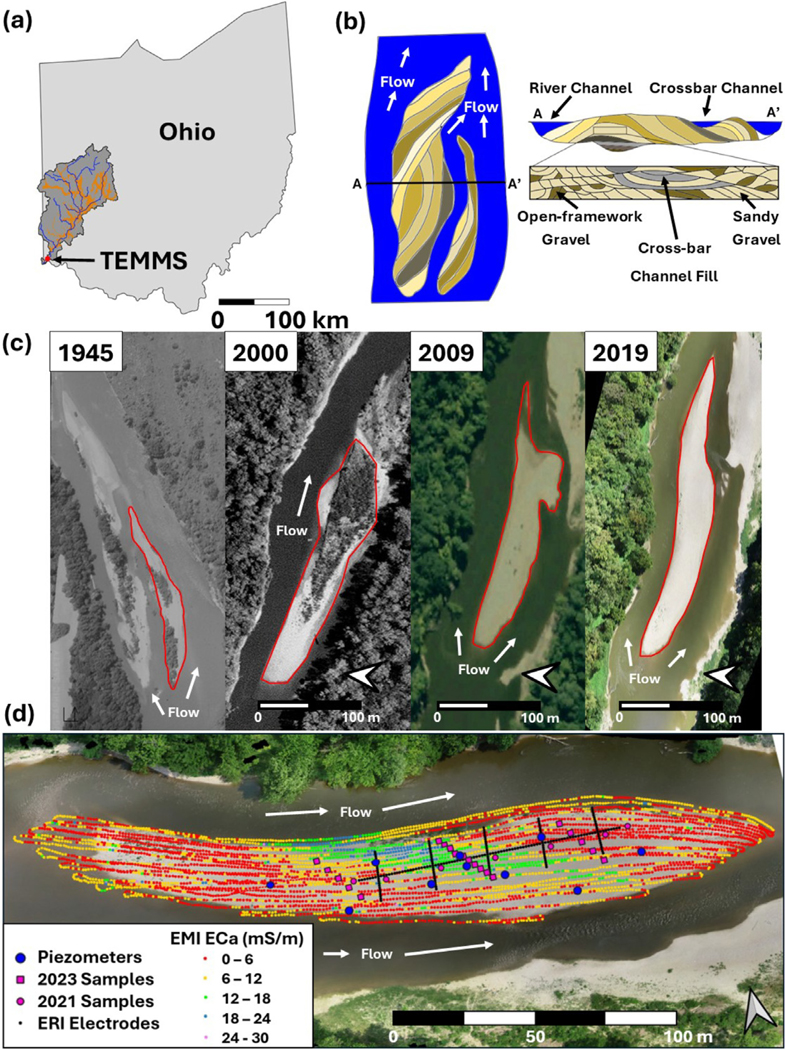
(a) Location of the Great Miami River watershed, Great Miami River Buried Valley Aquifer (orange), and the Theis-Nash Environmental Monitoring and Modeling Site (TEMMS). (b) Example compound bar depicting differing scales of geomorphological and sedimentary features. Nested within the scale of the entire compound bar is a mosaic of smaller-scale scroll bars, lobate bars, and cross-bar channel fills. Unit bar deposits consist of sets of cross-strata which are defined by differing sedimentary facies. (c) Historical imagery of the compound bar (outlined in red) at TEMMS in 1945, 2000, 2009, and 2019. 1945 imagery did not provide a scale or orientation and is rotated counter-clockwise 90° and cropped from the original image. 2000 and 2009 images were obtained from Google Earth. 2019 imagery captured via drone. Complete set of historical imagery can be found in the Supplementary Materials (Figure S1). (d) Compound bar at TEMMS. Displayed are the locations of piezometers (blue circles), sediment samples (pink squares and circles), ERI survey grid (black dots), and EMI survey points with measured apparent electrical conductivity values (ECa) at 1.5 m depth (red, yellow, green, blue, and purple dots). Drone imagery captured June 6, 2023.

Compound bars consist of scroll and lobate unit bars and cross-bar channel fills (e.g., filled former channels that crosscut a compound bar), and are controlling geomorphological features crucial to the dynamics of the hyporheic zone, particularly in gravel-bed rivers (see Fig. 1b). Their formation and evolution result from a combination of hydrological, sedimentological, and geomorphological processes, leading to complex and multiscale sedimentary architecture of varying grain sizes and compositions (Bridge and Lunt, 2006; Lunt et al., 2004, 2013). Previous studies provide insights into their development, sedimentary features, and influence on river channel morphology, emphasizing complex internal structures and their impact on sediment distribution and solute transport (Lunt et al., 2013; Rubin et al., 2006; Guin et al., 2010; Ramanathan et al., 2010). Lateral flow typically dominates the subsurface flow dynamics within compound bars, but enhanced downward flux occurs with higher magnitude flows (Wondzell and Swanson, 1999; Francis et al., 2010; Wroblicky et al., 1998). The significance of compound bars extends beyond their physical structure. Hyporheic flow through compound bar deposits facilitates biogeochemical processes critical to nutrient cycling and ecosystem health (Trauth et al., 2015; Marzadri et al., 2012; Groffman et al., 2005; Shope et al., 2012). Moreover, heterogeneity within compound bars, particularly within cross-bar channels containing finer, organic-rich sediments, creates unique microhabitats that contribute to the biodiversity of river ecosystems (Sackett et al., 2019; McGarr et al., 2021). Ultimately, compound bars play a significant role in solute mixing, nutrient transformations, and hyporheic exchange processes shaping hydrogeochemical dynamics and surface water-groundwater exchange.

Building upon previous foundational research (McGarr et al., 2021), this interdisciplinary study aims to unravel the complex interactions and intricate dynamics at play within compound bar deposits in riverine ecosystems and their influence on the hyporheic zone processes. McGarr et al. (2021) utilized geophysical methods such as electromagnetic induction (EMI) and time-lapse electrical resistivity imaging (ERI) to discern the relevant scales of heterogeneity within compound bar deposits. A key finding from their work was the identification of cross-bar channel fills as dominant contributors to the electrical signal. The cross-bar channel fills consisted of finer-grained facies with higher Fe, Al, and organic matter (OM) contents compared to the surrounding sandand gravel-dominated facies that predominated compound bar and riverbed alike. Similar findings have been found in other geophysical investigations (Fields et al., 2022). Motivated by these insights, our approach integrates various data sources and methodologies to offer a detailed exploration of the physical, chemical, and mineralogical aspects that govern these dynamic riverine environments. Our study has strategically installed piezometers within and outside of these cross-bar channel fills (Fig. 1d). This configuration, coupled with comprehensive sediment data, pore water chemistry analyses, and mineralogical studies, provides a multifaceted view of the role of cross-bar channel fills on hyporheic zone processes. These observations were designed to elucidate the role of cross-bar channel fills in shaping the hydrogeochemical dynamics within compound bar deposits. Building upon insights from other key studies (e.g., Shope et al., 2012; Sackett et al., 2019), this research brings to light the critical yet often underrepresented role of compound bars and fluvial islands in modulating surface water-groundwater exchange and their consequent impact on hydrological and biogeochemical dynamics.

## 2. Study site

The Great Miami River watershed spans approximately 9900 km^2^ in southwestern Ohio. It is hydrologically linked with the underlying Great Miami Buried Valley Aquifer (GMBVA), which is the sole source of drinking water for over 3 million people (Fig. 1a) (Wallace and Soltanian, 2021a). The GMBVA is cradled by paleovalleys consisting of interbedded Late Ordovician limestone and shale incised by pre-Pleistocene glaciation (Norris and Spieker, 1966; Teller, 1973). The glacial tills and outwash deposits that form the GMBVA were sourced by glacial outwash during the Illinoian and Wisconsinian glaciations 302 ka to 132 ka and 79 ka to 10 ka respectively (Fenneman, 1916; Norris and Spieker, 1966; Richmond and Fullerton, 1986; Dumouchelle, 1998). Most of the materials that compose the GMBVA are from the outwash plains of the Wisconsinian glaciation (Fenneman, 1916; Norris and Spieker, 1966). The modern riverbed consists largely of local limestone with granitic rocks of glacial provenance intermixed. Sediments from local shale also partially make up finer grained sediments (Brett et al., 2012; Waid and Brett, 2019; Bassarab and Huff, 1969).

The study site is a compound bar within the Great Miami River adjacent to the Theis-Nash Environmental Monitoring and Modeling Site (TEMMS) (39.2375°N, 84.7125°W) approximately 28 kilometers (km) upstream of the confluence of the Great Miami River and the Ohio River. At the TEMMS, the Great Miami River’s bank full channel width averages 150 m, with an average flow rate of 110 m^3^/s. The channel is generally incised, but the floodplain is periodically flooded during winter and spring peak flow conditions. The river stage fluctuates during storm events, ranging from as low as 0.5 m to nearly 8 m. The riverbed at the TEMMS is characterized by gravel-dominated compound and point bars consisting of planar and trough cross-bedded sands and planar cross-bedded gravels (Levy et al., 2011; Ritzi et al., 2002). The compound bar of interest is located between the main (north) and side (south) channels of the Great Miami River just north of the TEMMS floodplain. The compound bar has remained relatively stable despite its active evolution since at least 1945, evidenced by distinct differences and similarities when comparing current and historical imagery (Figs. 1c and S1). Historical imagery indicates fragmentation of a larger bar over the decades which would suggest the presence of complex sedimentary architecture today. This was confirmed with geophysical mapping by McGarr et al. (2021), also presented in Section 4.2, in which distinct zonation of the larger scale sedimentary architecture was identified. Based on the results in McGarr et al. (2021), a network of piezometers and stilling wells described further in Section 3.1 were installed in 2022 with locations determined by the results in McGarr et al. (2021) (Fig. 1d).

## 3. Methods

### 3.1. Hydrological monitoring

Changes in compound bar groundwater elevation and horizontal gradients were monitored by shallow piezometers within the compound bar; stilling wells were also installed in the river upstream and downstream of the compound bar to monitor stage changes (Fig. 1d). In the compound bar, 5-cm diameter PVC piezometers were installed to a depth of approximately 1.7 m. Each piezometer has a screen interval of 30.5 cm with solid casing extending to the surface. Stream temperature, intermittency and conductivity (STIC) loggers and pressure transducers were installed in each piezometer (Chapin et al., 2014). Piezometers PCB2, PCB4, and PCB7 were also instrumented with miniDOT dissolved oxygen loggers (Null et al., 2017). Periodic manual measurements of depth to groundwater and conductivity were used to convert pressure and STIC relative conductivity readings to groundwater elevation and specific conductance. The elevation of a marked location at the top of each piezometer and stage gauge was surveyed using differential-leveling referenced to the elevation datum of a local benchmark (Kenney, 2010). Measured groundwater elevations for sets of three piezometers that formed adjacent triangles across the network were used to calculate horizontal flow gradients using a three-point estimator calculation tool called 3PE (Beljin et al., 2013). STIC logger response was calibrated to standard salt solutions prior to deployment and checked following retrieval (Gillman et al., 2017). An initial configuration of piezometers across the compound bar included eight piezometers, three of which were installed within or immediately adjacent to the mapped location of a cross-bar channel that previously dissected the compound bar (PCB2, PCB4, PCB5) (see Section 4.2 and McGarr et al., 2021). Following an initial period of hydraulic monitoring, two additional piezometers were installed (PCB9, PCB10) to expand the network and provide locations that could be used to reliably monitor horizontal gradients through three-point estimation methods (Beljin et al., 2013) across the entire compound bar (see Figs. 6 and S5). Monitoring locations for measuring river stage were also constructed to support continuous measurements of water elevation and conductivity using pressure transducers and STIC loggers. The upstream river stage monitoring location at the start of the southern channel was also instrumented with a miniDOT logger to track dissolved oxygen.

### 3.2. Geophysical measurements

As part of the work presented in McGarr et al. (2021), electromagnetic induction (EMI) and electrical resistivity imaging (ERI) surveys were conducted. EMI survey was conducted on June 18th, 2020 (for results see McGarr et al., 2021) utilizing a DUALEM-421 (DUALEM Inc., Milton, ON, Canada) that consists of three pairs of dual-geometry sensors spaced at 1, 2, and 4 m (m) allowing for the collection of data at six depths of investigation (DOI). The two sensor alignments found within the DUALEM-421 are the horizontal co-planar array (HCP) and the perpendicular array (PRP). DOIs of 0.5, 1, 1.5, 2, 3, and 6 m are achieved as a function of the coil separation and sensor alignment (Triantafilis et al., 2011). Measurement locations were tracked using high precision, real-time GPS (kinematic Trimble R8s GNSS) to ensure a uniform distribution of data points during the survey. In total, 1114-point measurements were collected resulting in 6684 data points when considering all DOIs. Presented within this study are the EMI results of a survey with the same instrument model performed November 11, 2023 at a much higher spatial resolution (4745-point measurements resulting in 28,470 data points when considering all DOIs) also capturing the more recent morphology of the bar. The location, orientation, and spacing of the ERI grid was informed based on the cross-bar channel fills identified in the EMI data. The 211-electrode grid consisted of one 96-electrode line (L1-L1′) oriented roughly parallel to river flow and five 20 m spaced parallel 24-electrode lines (L2-L2′ - L6-L6′) oriented roughly perpendicular to river flow. Surveys were performing using an IRIS Syscal Kid 24 (IRIS Instruments, Orleans, France) with dipole–dipole arrays utilizing 1 m electrode spacing. The array and electrode spacing was selected to achieve a balance between DOI and spatial resolution (Ward et al., 2010). The DOI of all transects was approximately 5 m (1 to 3.8 m average thickness of compound bar deposits and channels fills reported in prior studies Lunt et al., 2004). Eleven ERI surveys were performed between July 11th, 2020 and August 10th, 2020 every third day river stage and weather dependent. During each survey, 801 measurements were taken across transect L1–L1′, while 153 measurements were taken across each parallel transect (L2–L2′, L3–L3′, L4–L4′, L5–L5′, and L6–L6′).

Fourteen ground penetrating radar (GPR) lines were collected across the TEMMS compound bar during August 2022. These included five lines parallel to the long axis of the bar and nine shorter orthogonal lines covering 1346 m total. Data were collected using a Sensors and Software Noggin SmartCart system borrowed from the EarthScope Primary Instrument Center (EPIC). Data were collected with a 250 MHz antenna and a 0.05 m step size. Data were collected with a 400 ps sampling interval over a 219.2 ns time window with 10 stacks. Positioning was acquired with an onboard GPS. All GPR data are archived with EPIC and are available at https://doi.org/10.7914/7214-h497 (Sturmer, 2022).

### 3.3. Geophysical data processing

EMI and ERI data were processed using the open source inversion packages EMagPy and ResIPy, respectively. In EMagPy (https://gitlab.com/hkex/emagpy), a cumulative sensitivity forward model (McNeill et al., 1980) was coupled with L-BFGS-B (Limited-memory Broyden–Fletcher–Goldfarb–Shanno with Bound constraints), a modification of the BFGS method (Byrd et al., 1995), which uses an estimate of the inverse Hessian matrix. The inversion problem was solved using the L1 norm objective function (McLachlan et al., 2021). EMagPy reports inverted results for bottom-of-layer depths (e.g., 0.5 m, 1 m, 1.5 m, 2 m, 3 m, and 6 m) based on the sensor spacing (see Section 3.2). Prior to inversion, EMI data were filtered based on apparent electrical conductivity (EC_a_) values (−10–35 mS/m) to remove erroneous measurements; 4657 of 4745 EMI measurements were used for inversion. The relative root mean squared error for inversion was 0.332 (Figure S2). ERI data were preprocessed using Prosys software (IRIS Instruments, Orleans, France), where raw data were filtered and prepared for inversion. Inversion was performed in ResIPy (https://gitlab.com/hkex/resipy) over a triangular mesh using the R2 inversion code (see McGarr et al., 2021 supplementary materials for details). Default settings within ResIPy were used for data inversion (Blanchy et al., 2020). The maximum number of iterations for inversion was set to 10, though most inversions converged after 2 or 3 iterations. All ERI data sets were filtered so that normalized inversion errors fell between ±3% (Binley et al., 1995) (see McGarr et al., 2021 supplementary materials). To examine the impact of hyporheic exchange on groundwater chemistry within the compound bar, the range of time-lapse ERI data was analyzed following the approach of Cardenas and Markowski (2011). For a more detailed description of the inversion methods used by EMagPy and ResIPy, see McGarr et al. (2021) and Blanchy et al. (2020), respectively.

GPR data were processed using EKKO Project^™^ V6 R1 GPR software (Sensors and Software, Mississauga, Ontario, Canada). Data were processed with filters including Dewow to remove low frequency noise and background subtraction to remove the strong air-surface interface at the tops of lines. Spreading exponential calibrated compensation gain was also applied to help normalize response from deeper reflectors. Reflection times were converted to depths using a measured velocity of 0.100 m/ns determined from hyperbola velocity calibration.

### 3.4. Physical and chemical sediment analysis

Sediment samples examined in this work encompass samples collected during two sampling campaigns: 7 samples used in McGarr et al. (2021) (B1–7) and 20 samples collected for ongoing work investigating contaminant fate and transport (US1–5, DS1–5, XB1–10). The 20 samples collected for contaminant fate and transport investigation were spatially located to parallel the approximate location of the cross-bar channel fills in the bar-tail/upstream portion of the bar (US1–5), in the bar-head/downstream portion of the bar (DS1–5), and within the cross-bar channel fills (XB1–10). One sample (DS5c) was a sub-sample of DS5 that was collected as a representative sample reflecting the fine grained material associated with cross-bar channel fills. As a result, the DS5 bulk sample was not analyzed as a whole. All samples were collected by digging boreholes with a gas-powered auger with a 0.3 m diameter bit to a depth of 100–150 cm (i.e., depth of borehole collapse), where substantial geophysical differences were observed in geophysical surveys. Sample locations from McGarr et al. (2021) corresponded to the centers of the lateral ERI transects (B2–B6), and at the bar-tail (i.e., the upstream end of the bar Lunt et al., 2004), hereafter termed (B1) and bar-head (i.e., the downstream end of the bar), hereafter termed (B7) ends of the longitudinal ERI transect. Grain size was quantified using traditional grain size analysis (ASTM E11) and the integral suspension method (Durner et al., 2017; Nemes et al., 2020). Oven-dried samples (100 °C for 24 h) were initially run through sieves ranging from 8 millimeters (mm) to 2 mm to eliminate cobbles and gravels. Sediments larger than 8 mm were weighed, measured (diameter), and categorized based on the Wentworth grain size classification. Samples <2 mm were then run through sieves ranging from 1 mm to 38 micrometers (μm). A separate aliquot of <2 mm sediments were also analyzed using the integral suspension method with an automatic particle size analyzer (METER Group PARIO) (Durner et al., 2017). Samples were prepared for loss on ignition (LOI) analysis by milling sediments in a SPEX 8000 mini mill using a pair of tungsten carbide balls and vial. Approximately 1 g of powder was used for LOI calculations. Samples were then processed through two more phases of baking at 550 °C and 1000 °C following standard LOI procedure for organic matter (OM) and carbonate content calculated using standard equations (Heiri et al., 2001). Powdered samples were then analyzed using a Bruker (Billerica, MA, USA) Tracer 5i energy dispersive portable X-ray fluorescence (pXRF). Samples were measured for 75 s and reported against the mudrock duo calibration from Bruker. For major elements, reported elemental weight% values were converted to oxide weight%. Data were also normalized and calculated using the LOI values. Three repeat analyses showed less than 5% variation for most major oxides and some trace elements, with higher % error (generally up to 20%) for elements with concentrations much less than one weight%. Mineralogy was determined by Mineral Labs Inc. (Saylersville, KY) using a Bruker D2 Phaser diffractometer with Cu radiation at 30.0 KV/10 ma. The scan was run over the range of 5°- 70° with a step size of 0.0167° and an accumulated counting time of 250 sec/step. The resulting diffraction patterns were analyzed with the aid of the Bruker Diffrac Suite. The identified crystalline phases where semi-quantitatively estimated with the aid of the Topaz software.

### 3.5. Water sample collection and geochemical analysis

River stage and weather permitting, water samples were collected every third day between July 22, 2022 and August 22, 2022 from piezometers PCB1–8 and the river. Pore water samples were collected using a stainless steel push point sampler (MHE Products, East Tawas, MI, USA) to reach the screened interval of the piezometer attached to a peristaltic pump (Solinst, Georgetown, ON, Canada). Prior to sampling, water was pumped through a YSI ProDSS flow cell (Yellow Springs, OH, USA) and readings of temperature, pH, dissolved oxygen (DO), and specific conductance were recorded after approximately 5 min when parameters stabilized. Water samples collected for ferrous iron were processed on-site using a HACH (Loveland, CO, USA) portable spectrophotometer with 1,10-Phenanthroline color reagent and Method 8146 (Trutschel et al., 2022). Water samples collected for Ion Chromatography were passed through a 0.2 μm filter and were kept on ice or at 4 °C until analysis. Samples were analyzed with a Dionex Aquion Ion Chromatograph (Thermo Fisher Scientific, Waltham, MA, USA) using a Dionex IonPac^™^ AS23–4 μm column under manufacturers protocol for anions (F^−^, Cl^−^, NO^2−^, Br^−^, NO^3−^, PO_4_^3−^ and SO_4_^2−^) and Dionex IonPac^™^ CS12 A column under manufacturers protocol for cations (Li^+^, Na^+^, NH_4_^+^, K^+^, Mg^2+^ and Ca^2+^) (Trutschel et al., 2023). Water for dissolved organic carbon (DOC) analysis was collected in acid-washed, amber glass vials, acidified in the field to pH 2, and analyzed on a Shimadzu TOC-V total organic carbon analyzer via non-purgeable organic carbon method.

### 3.6. Digital topography from drone structure-from-motion

To characterize riverbed, bank, and bar topography at bedform-resolving scale digital elevation models (DEMs) and orthoimagery were generated using standard structure-from-motion (SfM) photogrammetry techniques (e.g., James and Robson, 2012). For each survey, 20-megapixel aerial images were collected using a DJI Phantom 4 Pro UAV. About 850 photos were collected per flight, covering the full width of the river along a 1.2 km reach, at a typical altitude of 40–50 m above ground level. These were processed using AgiSoft Metashape software (v1.6.3) to 2–3 cm resolution digital elevation model grids. Eight surveys were collected between September 2019 and June 2023, including five flights at shorter intervals during the water sampling period in July–August 2022. Georeferencing and camera calibration were controlled by twelve persistent ground control points (GCPs) measured using a Trimble RS8 realtime kinematic GPS system. Between 2022 and 2023 some of the rebar GCP markers were lost due to sedimentation and/or human activity, so new points were established, and additional persistent topographic markers (e.g., tree centers) were defined to improve fine alignment across all surveys. The spherical point alignment accuracy of the grids were estimated to be approximately 20 cm. Shorelines were delineated using GIS software (QGIS, v3.28) to trace the water’s edge in the orthoimagery from each survey at a map scale of 1:250.

## 4. Results

### 4.1. Physical and chemical sediment analysis

**Fig. 2.**
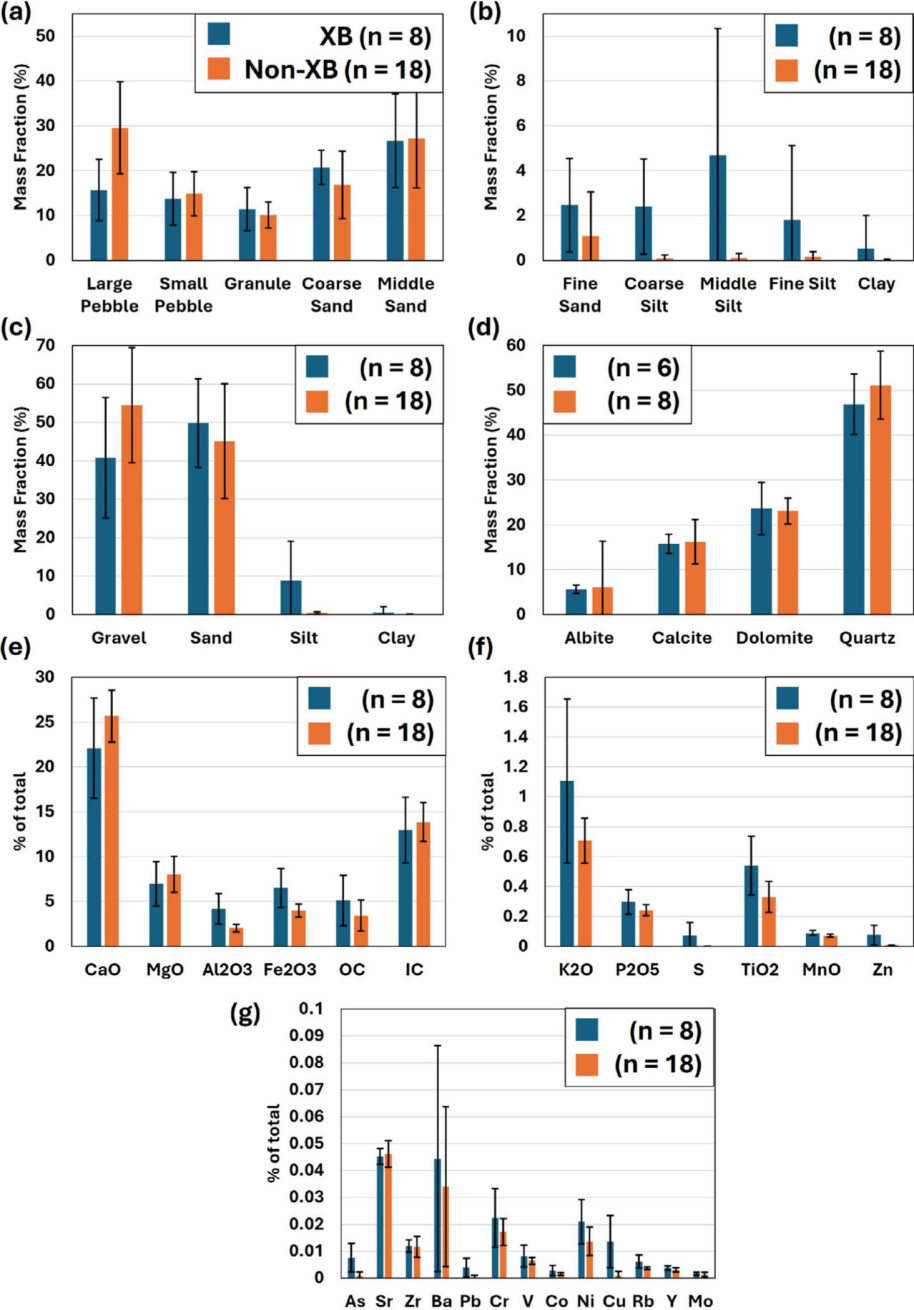
Comparison of sediments from cross-bar channel fills (XB) represented by blue bars and other sediments (Non-XB) represented by orange bars. Average values of grain size analysis of sediments (a) > 200 μm, (b) < 200 μm, and (c) cumulative. (d) Average values of mineralogy (>5 weight percent). Average values of bulk element oxide content of (e) >2 weight percent, (f) 2–0.05 weight percent, and (g) <0.05 weight percent. In (e), organic matter content (OC) and carbonate content (IC) are also visualized. SiO_2_ is not depicted to better accentuate the differences of lower weight percent elements (XB contains 46% and Non-XB contains 48%).

Samples examined in McGarr et al. (2021) (e.g., B1–7), as well as the more recent samples (e.g., US1–5, DS1–5, and XB1–10) all revealed a distinct zonation in sedimentary facies spatially across the bar that corresponded with observations in geophysical surveys discussed further in Section 4.2. Analysis of sediments presented here was performed using a combination of traditional sieves and the integral suspension method (Fig. 2a–c). In boreholes corresponding to sample locations B1–7, samples were taken at numerous depths that all displayed a fining downward trend. In B1, B2, B5, B6, and B7, each sample was dominated by gravels and sands with less than 1% of sediments <0.1 mm. Analysis of samples US1–5, DS1–4, XB3, and XB8–10 revealed similar results. In boreholes B3 and B4, samples taken below 70 cm and 100 cm, respectively, consisted of 20%–30% <0.1 mm grains which are identified as a cross-bar channel fill. Analysis of samples XB1, XB2, and XB4–7 also contained higher silt contents; however, these samples consisted of only 2%–6% <0.1 mm suggesting they are potentially from the edge of the cross-bar channel fill deposit. Sample DS5 also contained finer grained material, but in this case the finer grained material (exists as chunks of a similar consistency to modeling clay in all samples from within the cross-bar channel fill deposit (see Figure S4)) was separated from the bulk sample for analysis. When examining the <2 mm fraction of DS5c (fine grained sub-sample of DS5), sediments consisted of 74% sand (38% fine sand 0.2–0.05 mm compared to <5% in all other samples), 16% silt, and 8% clay.

XRF and LOI results for sediments <2 mm also displayed a similar zonation across the bar with apparent differences between cross-bar channel fill sediments and the sands and gravels that dominate the bar (Fig. 2e–g). Cross-bar channel fills contain 5.1% (σ = 2.8) organic matter and 13.0% (σ = 3.6) carbonate. The gravel- and sand-dominated sediments contain 3.4% (σ = 1.7) organic matter and 13.9% (σ = 2.1) carbonate. Based on differences in grain size distribution and observations of those grains, the disparity between carbonate content would be larger if gravels were considered in this analysis as most of those are limestone. XRF results also revealed spatial variability in terms of weight percentage elemental composition. Differences in elemental composition were confirmed with an equal variance two tail t-test with a 0.05 *p*-value indicating similarity or difference between the samples. SiO_2_ across all samples was similar with cross-bar channel fills consisting of 39.8% (σ = 4.6) and gravel- and sand- dominated sediments consisting of 41.5% (σ = 4.6) (*p*-value = 0.39). Cross-bar channel fills contained higher weight percentages of Al_2_O_3_ (4.2%, σ = 1.7), Fe2O3 (6.5%, σ = 2.2), and TiO2 (0.5%, σ = 0.2) and lower CaO (22.1%, σ = 5.6) and MgO (7.0%, σ = 2.5) compared to the gravel- and sand-dominated sediments (Al_2_O_3_: 2.0% (σ = 0.4), Fe_2_O_3_: 4.0% (σ = 0.7), TiO_2_: 0.3% (σ = 0.1), CaO: 25.7% (σ = 2.9), MgO: 8.0% (σ = 2.0)). The t-test confirms most of these observations with Al_2_O_3_ (*p*-value = 3.19E−5), Fe_2_O_3_ (*p*-value = 0.0001), TiO_2_ (*p*-value = 0.001), and CaO (*p*-value = 0.04) being statistically different and MgO (*p*-value = 0.25) being statistically similar. DS5c, isolated fine grained materials from a cross-bar channel fill sample, consisted of 39.2% SiO_2_, 7.4% Al_2_O_3_, 11.1% Fe_2_O_3_, 0.9% TiO2, 15.25% CaO, and 4.1% MgO.

Minerals for sediments <2 mm that make up >5% of samples (e.g., quartz, calcite, dolomite, and albite) in both cross-bar sediments and the rest of the compound bar are quite similar with weight percentages in both being separated by <1% other than quartz, where cross-bar channel fills consist of 5% less (Fig. 2d). Quartz and limestone minerals make up the majority of the samples with 45%–50% weight percent quartz, 16% calcite, and 23% dolomite. The differences between sediment mineralogy across the bar lies primarily within the detrital and clay minerals. These minerals are not presented within Fig. 2 because many are observed only in one sample. Two forms of muscovite (muscovite: 5.27% in US1 and 4.94% in DS3; 2m1 muscovite: 4.4% in XB8) are found within the gravels and sands that were not detected in the bulk cross-bar channel fill sediments. Kaolinite, richterite, glauconite, and clinochlore are all present only within the cross-bar channel fills. In samples where these minerals were detected, they occur at weight percentages from 2.14% to 5.68%. Glauconite was the most commonly detected of these minerals in cross-bar channel sediments with three of six samples reported to contain glauconite (2.61%, 4.44%, 5.68% in XB2, XB5, and XB7, respectively). Clinochlore was detected in two cross-bar channel sediment samples (2.14% and 3.5% in XB6 and XB7, respectively), and richterite (3.75% in XB1) and kaolinite (3.9% in XB5) were detected in one. DS5c (isolated fines) consists of 11% calcite, 16% dolomite, 35% quartz, 28.35% 2m1 muscovite, and 9.7% kaolinite.

### 4.2. Geophysical imaging

**Fig. 3.**
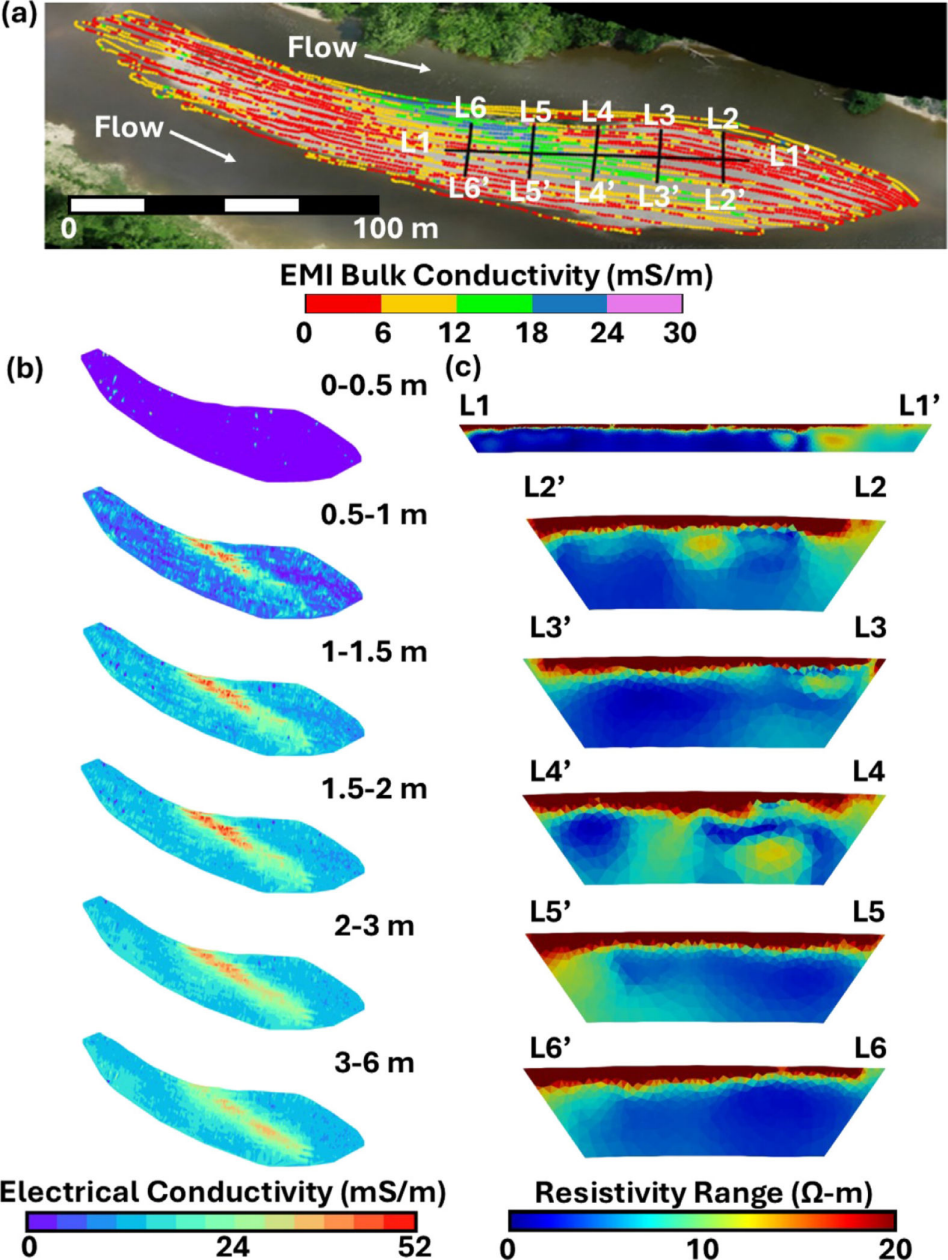
(a) Aerial image of TEMMS compound bar with EMI bulk conductivity measurements at 1.5 m depth and ERI grid location. (b) Inverted EMI results for layer depth corresponding to DUALEM depths of investigation. (c) Range of electrical resistivity change as measured across all survey dates. Range of the uppermost portion (approximately 1 m) is higher than the scale captures as large resistivity changes were observed due to large changes in saturation. Resistivity ranges larger than 11 Ω-m are indicative of hyporheic mixing following Cardenas and Markowski (2011). All transects have depths of investigation of approximately 5 m. L1-L1′ is 96 m long. All other transects are 24 m long.

Raw EMI electrical conductivity data (ECa), inverted EMI data, and inverted ERI data (initially reported in McGarr et al. (2021)) all show distinct zonation across the compound bar that aligns with differences in sedimentary architecture in the subsurface (Fig. 3). An area that runs sub-parallel to the river channels is evident, which corresponds to cross-bar channel fills. The differences in sediment characteristics were presented in the previous subsection, and their influences on geochemistry and hydrology will be explored further in following subsections. In short, cross-bar channel fills represent a former comparatively low energy depositional environment where finer grained materials were allowed to settle out of the water column that today is now an area with distinct sedimentary characteristics that drive a differential geophysical response.

EMI results from different DOIs reveal both differences in pore saturation as well as sedimentary architecture (Fig. 3b). Within the uppermost meter, a weak electrical response (0–10 mS/m) occurs that is caused by surficial cobble- and gravel-sized sediments underlain by unsaturated gravels and sands. At base flow conditions when the EMI survey was performed, the water table was at approximately a meter below ground surface as seen during the sediment sampling process described in Section 3.4. In every layer other than 0–0.5 m, non-zero (>0 mS/m) values are reported indicating the presence of pore water with partial saturation occurring in the 0.5–1 m layer and full saturation occurring in each subsequent layer. Also apparent within each layer beneath 0.5 m is a zone of higher electrical conductivity (30–60 mS/m) that corresponds to the finer grained cross-bar channel fills. Sediments in the lower electrical conductivity areas of the bar are predominantly gravels and sands that make up the majority of the surrounding riverbed.

ERI results initially reported in McGarr et al. (2021) tell a similar story with three distinct zones of electrical resistivity that agree with the EMI survey results. Along each ERI survey transect, the uppermost meter exhibits relatively high electrical resistivity values (> 107 Ω-m) indicating unsaturated sediments. Similar to the EMI survey, beneath the unsaturated zone there are two distinct electrical resistivity signals. The central portion of L1-L1′ and variable portions of L3-L3′, L4-L4′, L5-L5′, and L6-L6′ exhibit a zone of low electrical resistivity (10–20 Ω-m) that indicates the finer grained cross-bar channel fills (Fig. 3). The remainder of each transect displays a higher electrical resistivity that indicates gravel- and sand-dominated sediments; however, when comparing the bar-head to the bar-tail portions of these sediments there is a differing electrical response. The variability in electrical response is driven by the gravel to sand ratio where the bar-tail (33–66 Ω-m) has a lower gravel content than the bar-head (66–107 Ω-m). McGarr et al. (2021) examined the relationship between median grain size and electrical response and observed correlation with a power function where decreasing grain size resulted in lower electrical resistivity. Ultimately, it is apparent when comparing the EMI results and location of the ERI grid that the responses of each survey technique align spatially.

Time-lapse ERI results captured the impact of sediment heterogeneity on hyporheic mixing (Fig. 3c). McGarr et al. (2021) identified that an electrical resistivity range of > 11 Ω-m during the survey period at this site was evidence of hyporheic mixing. The range of resistivity is driven by changes in pore water chemistry over the survey period. When examining the saturated portion of the bar (over 1 m beneath ground surface), the low electrical resistivity cross-bar channel fills exhibit a resistivity range of less than 2 Ω-m which indicates stable pore water resistivity throughout the survey period (McGarr et al., 2021). This would indicate that there is likely limited hyporheic mixing occurring within this zone that also suggests limited groundwater flow and hyporheic mixing through this zone as explored further in Section 4.4. Outside of the cross-bar channel fills, the bar-tail was more stable than the bar-head with resistivity ranges of 1 to 4 Ω-m and 1 to 13 Ω-m, respectively (McGarr et al., 2021). This indicates that there is more variable pore water conditions within the gravels and sands suggesting increased flow and connectivity to the river. This is undoubtedly the case in the bar-head where the resistivity range values cross the threshold of 11 Ω-m indicating hyporheic mixing.

**Fig. 4.**
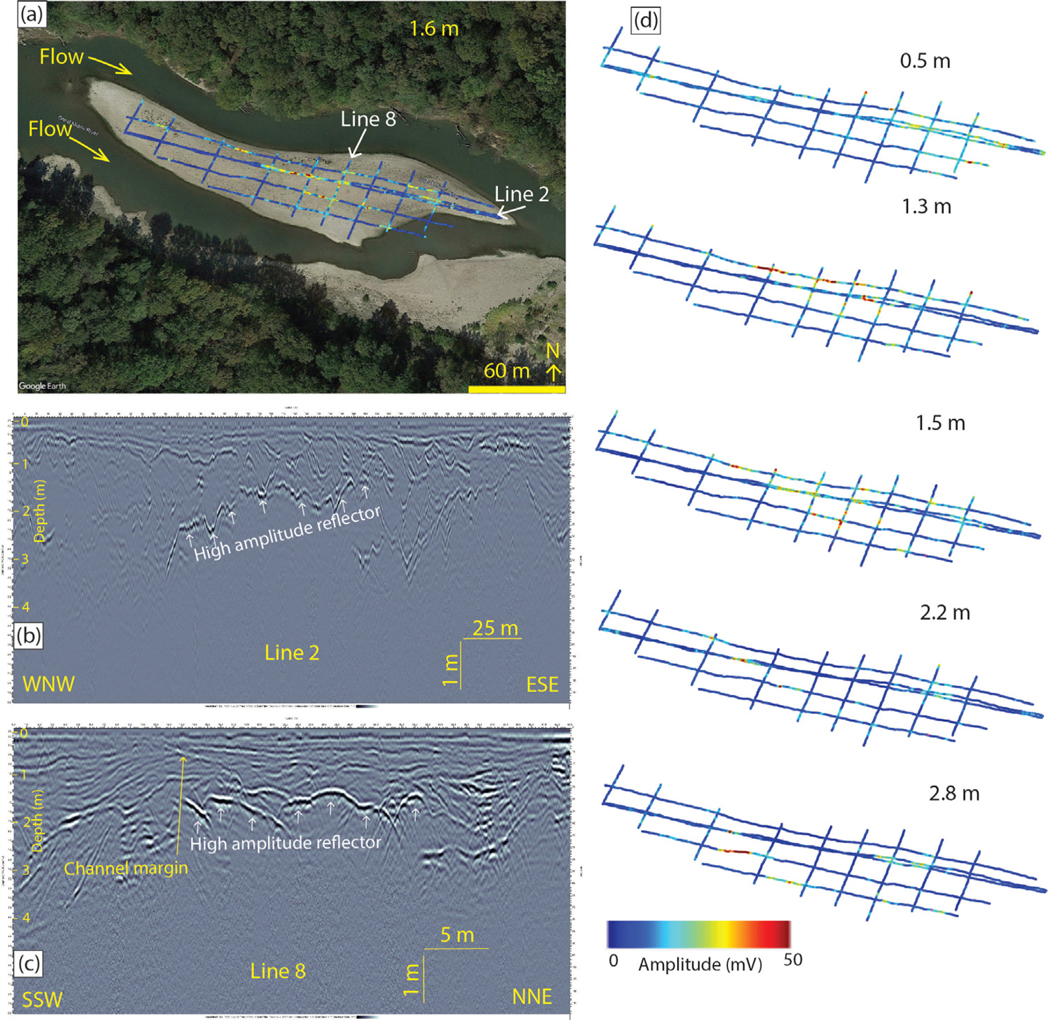
Representative GPR data from compound bar. (a) Amplitude data for 1.6 m depth projected on a surface Google Earth image. Note that the water level was lower during the survey. Positions for lines in 2 and 8 also noted. (b) Cross-sectional profile for line 2. Note the high amplitude reflector in the central part of the line that trends downward towards the WNW. (c) Cross-sectional profile for Line 8. (d) Amplitude depth slice maps for five depths. North is up on all profile maps. As depth increases the high amplitude reflections sweep towards the SW delineating a NW-trending feature within the compound bar.

GPR data are consistent with results from EMI and ERI studies with respect to channelization and higher presence of finer-grained material throughout the central portion of the bar. The GPR lines show reflectors down to 3–4 m depth. The lines have inclined reflectors that truncate other sets of reflectors, interpreted as cross-bed sets within the compound bar (Fig. 4). Most of the reflectors are relatively low amplitude with the exception of a few higher amplitude reflections present in the central part of the bar. The higher amplitude reflections generally sweep southwestward across the central part of the compound bar from depths of 1–1.4 m in the northeast to 2.4–3 m in the southwest, tracing out a broad northwest-trending channel (Fig. 4b–d). The high-amplitude response within the central portion of the bar is interpreted here as being generated by a strong reflectance from a contact between finer-grained material rich in silt and clay and coarser-grained material that comprises the bulk of the compound bar. Lower amplitude reflections are interpreted as representing bedding lithology changes with lower reflection coefficients such as boundaries between saturated sandy and silty beds. The interpretation of the highly reflective surfaces as contacts in the central portion of the channel as representing presence of fine-grained material in contact with coarser material is consistent with the observations of a northwest-trending zone of high electrical conductivity observed at depths of 1 to 6 m through this portion of the compound bar in McGarr et al. (2021) and discussed above (McGarr et al., 2021).

### 4.3. Geomorphology

**Fig. 5.**
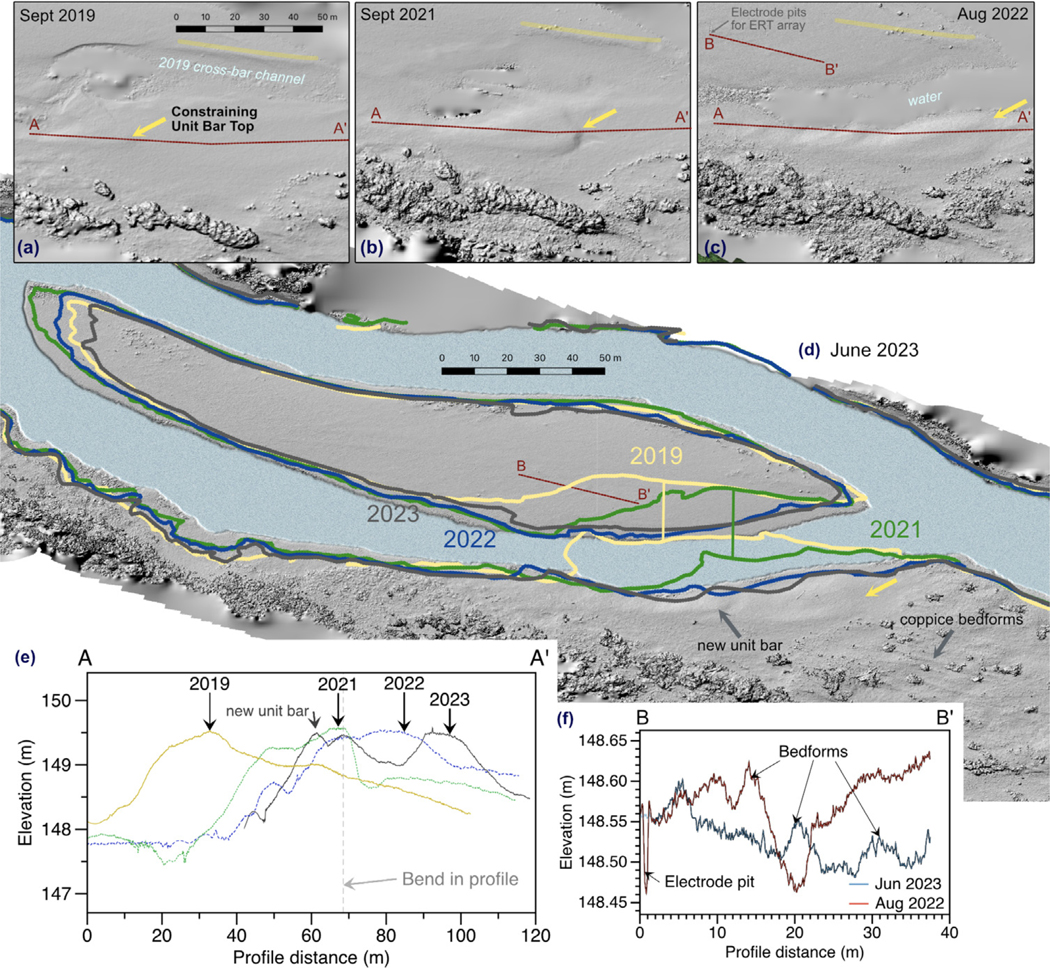
Geomorphic changes,2019–2023. (a–d) Hillshaded Structure-from-Motion (SfM) DEMs with baseflow shorelines by year. Migration of a 1.5-m tall unit bar (yellow arrow) corresponds to change in position of the cross-bar channel. (e) Elevation profile showing unit bar migration along A-A′. (f) Changes to decimeter-scale bedforms suggest a 20–30 cm active layer during typical floods. Note that the SfM reconstructions resolve bathymetry to about 10 cm water depth.

Repeat drone surveys facilitated examining interannual changes to topography of the riverbed and compound bar (Table 1). The most prominent change to the bar topography is the downriver translation of the cross-bar channel by a full channel width from 2019–2022 (Fig. 5). Adjustment of the cross bar channel is paced by migration of a 1.5-m tall unit bar that defines its outer bend, the front of which moved 80 m over four years. During large floods, as the unit bar moves closer to the higher, vegetated portion of compound bar, more resistance to flow in this region steers the water back towards the center of the main channel. By 2023, migration of this bar slowed and the bedform became more parallel to the cross-bar channel. In addition, a new bar front of similar dimensions began to emerge at the cross-bar channel bend in 2022, following a similar trajectory. The period of highest river stage (typically in February or March) completely submerges the upper vegetated part of the bar near the south (right) bank and enables bedload transport on a more directly downriver vector (e.g., coppice bedforms visible in Fig. 5d). On the bar tops, changes to decimeter-scale bedforms suggest 1–3 m per year migration and indicate a 20–30 cm active layer during typical floods.

### 4.4. Hydrology

**Fig. 6.**
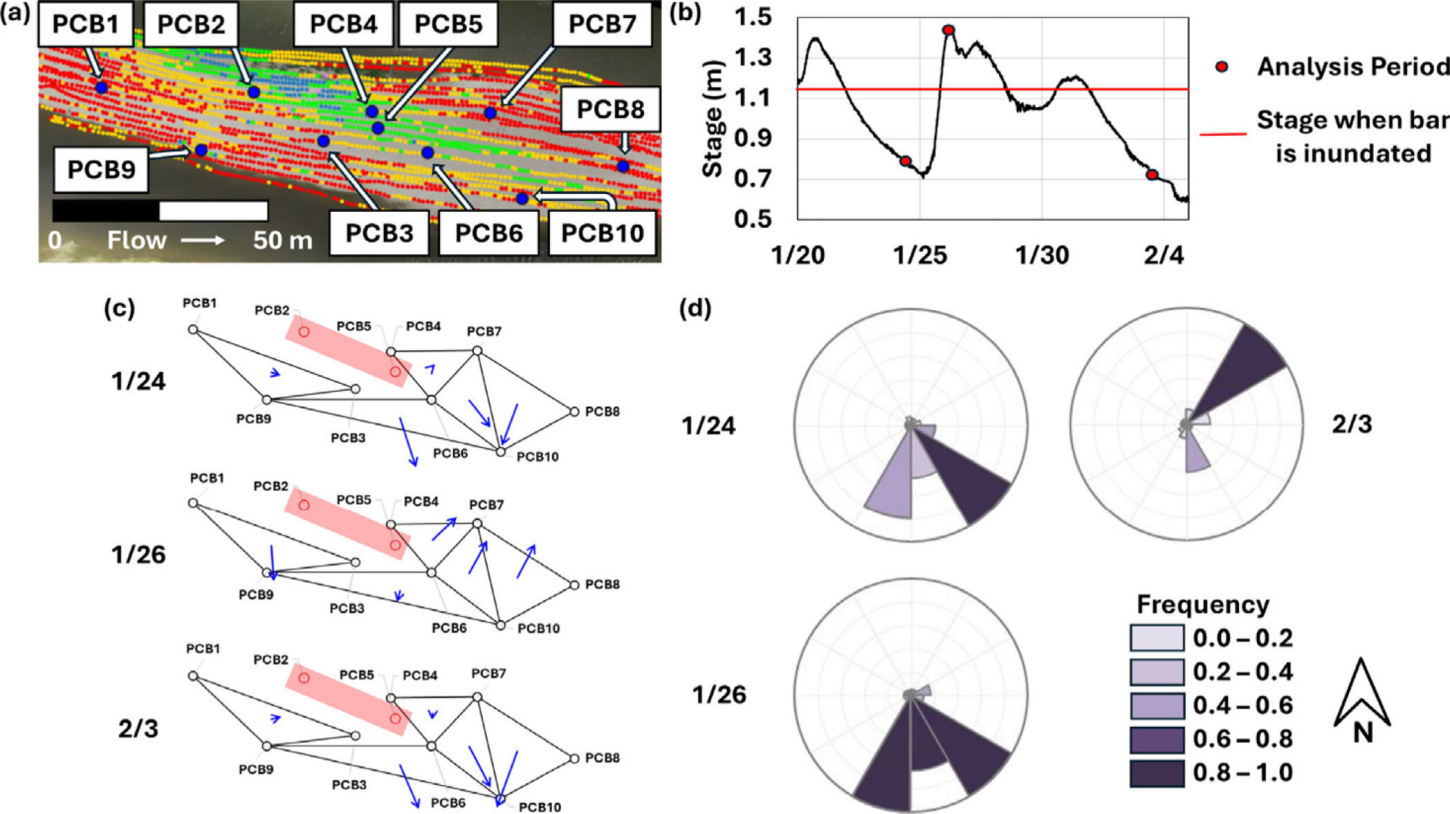
(a) Aerial image of TEMMS compound bar with piezometer locations. (b) River stage during the period examined in this figure which covers two inundation cycles of the compound bar. Red dots indicate analysis dates (1/24/23, 1/26/23, and 2/3/23). The red line indicates when the bar is inundated at approximately 1.2 m river stage. (c) Daily average flow gradients for 1/24/23, 1/26/26, and 2/3/23 developed using 3PE. The red rectangle (including PCB2 and PCB5) represents cross-bar channel fills which are a baffle to flow; prevents use of 3PE triangles that encompass this hydrologic discontinuity. (d) Rose diagram representing gradient directions across the bar for 1/24/23, 1/26/26, and 2/3/23.

As discussed in Section 3.1, eight piezometers were originally installed across the compound bar, three of which (PCB2, PCB4, PCB5) were installed within or immediately adjacent to the mapped location of the cross-bar channel fill (Fig. 6a). Following the initial monitoring period, the piezometer network was assessed to determine locations with geologic discontinuities (Figs. 6a,c, S5, and S6). The sluggish response of groundwater levels at PCB5 following sampling events (Figure S6), along with the fine-grained lithology of sediments across the area containing piezometers PCB2 and PCB5, indicated a discontinuity in the flow field across this area. This type of flow discontinuity indicated that piezometers PCB2 and PCB5 should not be used to estimate hydraulic gradients. Based on these analyses, additional piezometers PCB9 and PCB10 were installed to better monitor ground-water flow based on compound bar hydrostratigraphy (Figure S5). Re-examination of hydraulic gradient data with the updated piezometer network demonstrated that flow vectors deflected around the area containing PCB2 and PCB5, consistent with the hydrostratigraphy data (see red rectangle in Fig. 6c). These observations indicated that 3PE triangle PCB3-PCB4-PCB6 was inappropriate due to discontinuous geology within the cross-bar channel fill.

The *K* at a subset of piezometers (PCB2, PCB4, PCB5) was estimated using their respective hydraulic response to pumping during collection of samples for analysis (Figure S7). The drawdown during sampling deviates from the head change induced for a conventional rising-head slug test, but the change in head as a function of time during recovery can be used to estimate relative *K* for each location. The Darcy solution for a falling-head permeameter test reported by Landon et al. (2001) was used to calculate *K* for streambed sediments (Landon et al., 2001). This relationship uses the head change over time relative to the depth interval of the sediment being tested to estimate *K*. For this calculation, the sediment depth interval was set to the distance between the surface of the compound bar to the middle of the screen interval for each piezometer (PCB2 169.6 cm, PCB4 169.2 cm, PCB5 171.95 cm) and used the head change between sampling events recorded by the logging transducers in each piezometer. There were 14, 4, and 17 events with usable data for piezometers PCB2, PCB4, and PCB5 with an average estimated *K* of 0.30, 1.1, and 0.14 cm/d, respectively. For locations PCB2 and PCB5, the casing water elevation did not fully re-equilibrate to a stable level between most of the sampling events, so these estimated averages are likely biased high. Examination of the change of head record at each location indicated that the casing water elevation returned to a static level after about 6 and 32 days for PCB2 and PCB5 with estimated *K* of 0.2 cm/d and 0.02 cm/d, respectively (Figure S8). The hourly transducer logging interval was too coarse to adequately characterize the water table rebound rate at PCB4, but the maximum calculated *K* was estimated as at least 1.3 cm/d. A previous study further north in the watershed presented the results of *K_h_* for “riverbed” slug tests at depths of 0.357–1.52 m ranging from 1.1 to 78.0 m/d. These values are potentially representative of locations outside of the cross-bar channel fills (Levy et al., 2008). The water elevation in piezometers indicates hydraulic connectivity between the river and the groundwater, with the piezometers responding to changes in the river level. PCB2 and PCB5 exhibit a delayed response to stage change in the Great Miami River (Fig. 8) that aligns with the interpretation of cross-bar channel fills exhibiting lower *K*. PCB4 is more closely aligned with river stage indicating this piezometer is located on the fringes of the cross-bar channel fills where it is both hydraulically connected to the river and hydrogeochemically (see Section 4.5) connected to the cross-bar channel fills.

**Fig. 7.**
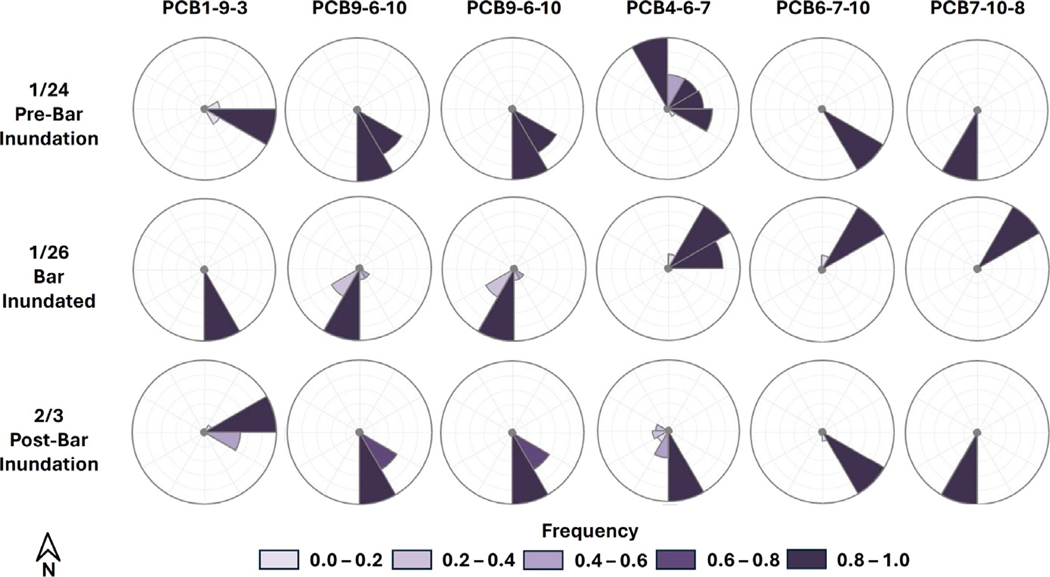
Rose diagrams generated with 3PE showing flow variability during river stage fluctuation resulting in inundation of the TEMMS compound bar also shown in Fig. 6. Each row corresponds to a date and period of river stage when the bar is either inundated or not inundated. Each column corresponds to a flow diagram triangle seen in Fig. 6c.

**Fig. 8.**
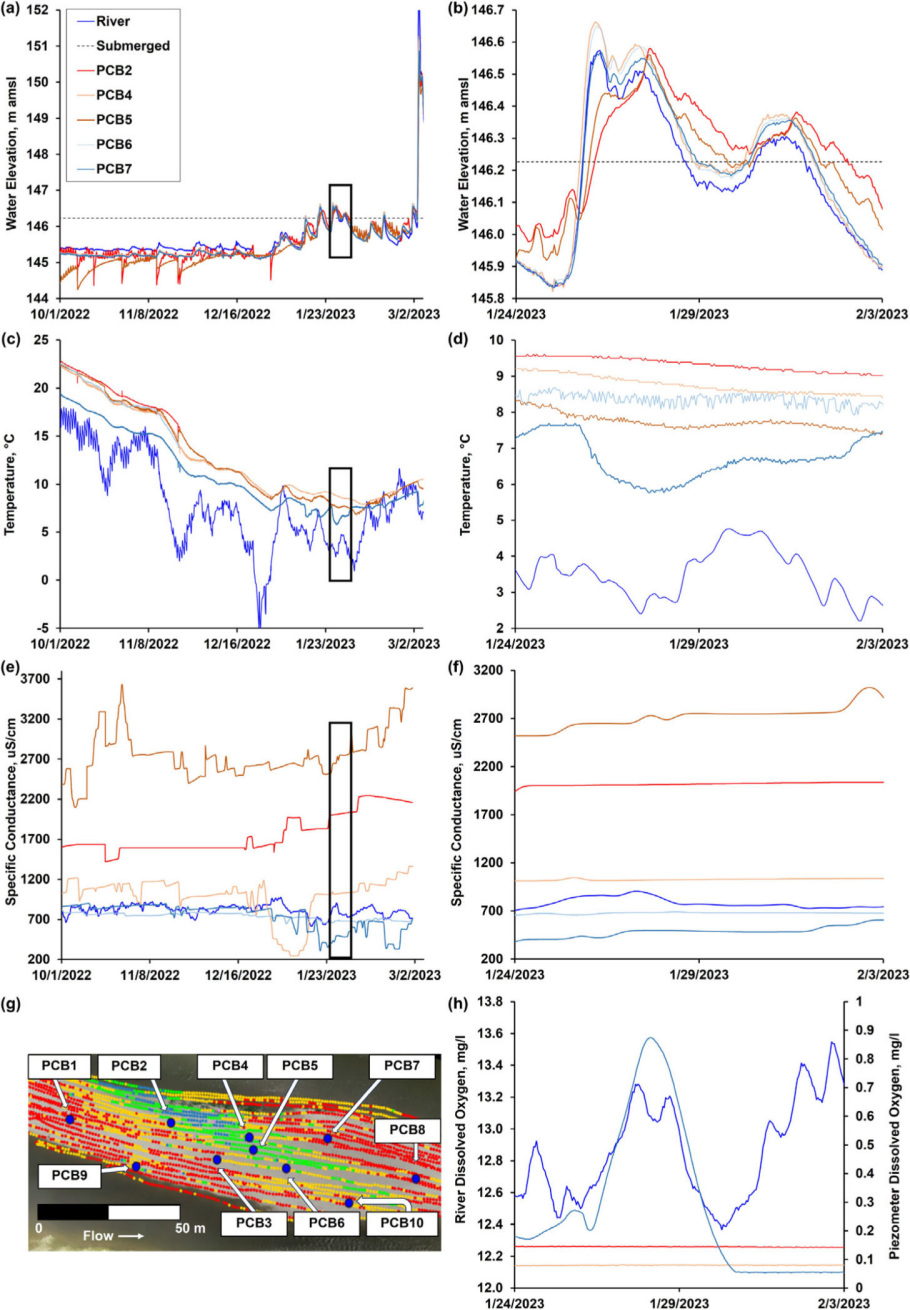
(a, b) Water level elevation (m amsl), (c, d) temperature (°C), (e, f) specific conductance (uS/cm), and (h) dissolved oxygen (mg/L) in both river water and groundwater across the (g) piezometer network depicted at the bottom left of the figure. The left side of the figure includes data from October 1st 2022 to March 2nd 2023. The right side of the figure includes data from January 24th 2023 to February 3rd 2023. This subset of time (see boxes in Fig. 8a, c, e) was selected as it represents a period where piezometers were sealed and no sampling events occurred introducing potential disturbances to data collection. Dissolved oxygen is only displayed for this period.

Between January 24, 2023 and February 3, 2023, the compound bar went through a cycle of inundation over which the hydrologic response was monitored (Figs. 6, 7, 8). The hydraulic gradient direction and magnitude for each monitoring interval was estimated using the 3PE calculation tool (Beljin et al., 2013). When interpreting 3PE triangles, it is important to note that the gradients map the potential for flow, but the actual flow directions will be dictated by other factors in addition to hydraulic head distribution (Fig. 6c). On January 24, 2023 and February 3, 2023 (pre-inundation and post-inundation), the majority of 3PE flow triangles report flow towards the southern (side) channel. The directions point to an area in the southern channel with a sharp decrease in channel bottom elevation (see riffles in Fig. 1d). Under base flow conditions, this discontinuity in channel morphology results in lower river stage elevation in the southern channel relative to the northern (main) channel, which drives groundwater flow gradient in a southern direction across this part of the compound bar. On January 26, 2023 (bar inundated) 3PE flow triangles on the downstream end of the bar flip their gradients towards the northern (main) channel. When the compound bar in this area is inundated, it appears that groundwater flow direction is driven by the overall direction of river flow. In all flow conditions, all 3PE flow triangles do not report flow into the cross-bar channel fills, which confirms that this zone is a baffle to flow due to the finer grained low *K* sediments present.

### 4.5. Hydrogeochemistry

Comprehensive analyses of river and groundwater water samples from PCBs 1–8 suggest a positive relationship between temperature variations and biological activity and reaction rates within the compound bar. This is consistent with previous work showing temperature trends following a seasonal pattern are expected to affect the chemistry and biology of the groundwater system, including reaction rates and microbial activity (Von Gunten et al., 1991; Riedel, 2019). Specific conductivity data varies significantly between sample locations (Table 2), indicating variations in ionic content caused by geochemical inputs or processes such as mineral dissolution. Namely, elevated specific conductivity readings within the cross-bar channel fill sediments (e.g., PCB2 and PCB5) coincide with high levels of DOC, Fe^2+^, and NH_4_^+^. Groundwater and surface water pH, which can influence and be influenced by microbial activity and geochemical reactions, also varies from neutral (pH = 7.1) to alkaline (pH = 8.7). This is particularly relevant considering the varying levels of electron acceptors (e.g., NO_3_^−^, NH_4_^+^) and donors (e.g., DOC) across the compound bar, which often indicate variable redox zonation and potential microbial hotspots. Low dissolved oxygen (DO) levels across the compound bar further suggest the occurrence of anaerobic conditions conducive to microbial processes such as denitrification or sulfate reduction.

**Fig. 9.**
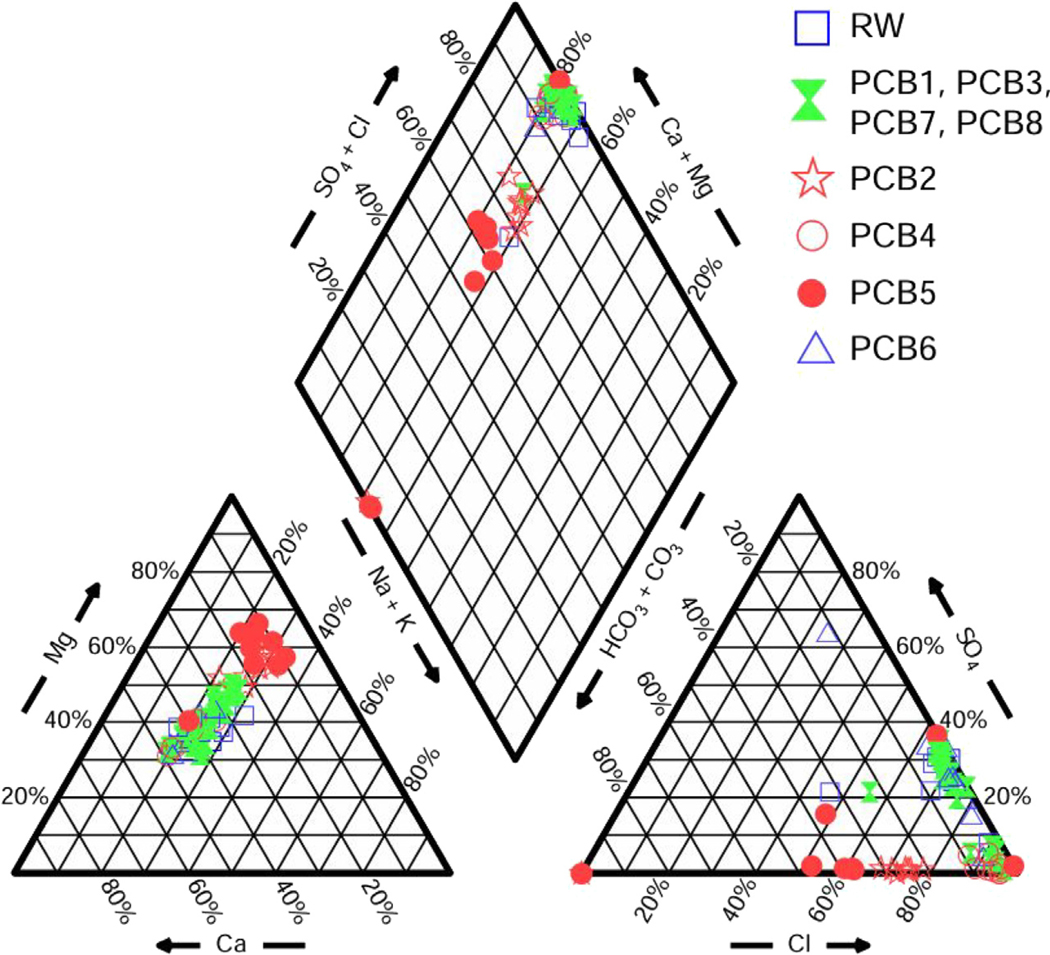
Piper diagram of geochemical data with red shapes showing data from PCB2, PCB4, and PCB5 and all other shapes showing data from the remaining piezometers and the river. PCB2 and PCB5 are located within the cross-bar channel fill. PCB4 is located in a transitional zone between the cross-bar channel fill and the rest of the bar that is more hydraulically connected to the river.

Principal component analysis (PCA) and Piper diagrams provide a fingerprint of groundwater chemistry, highlighting the distinct geochemical profiles of locations within the cross-bar channel fill (e.g., PCB2, PCB4, and PCB5) (Figs. 9 and S9). Varying DO levels imply variable redox conditions and the potential for both aerobic and anaerobic microbial processes. High concentrations of DOC in piezometers within the cross-bar channel fill (e.g., PCB2, PCB4, and PCB5) suggest increased organic matter input from sediments. Ammonium levels highlight the ongoing processes within the nitrogen cycle, possibly indicating zones of mineralization and nitrification. When considering data from across the bar, it becomes evident that there are unique geochemical niches within the groundwater system, characterized by the stable input of DOC influenced by surface water-groundwater interactions.

## 5. Discussion

Compound bars exhibit distinctive and often complex sediment heterogeneity that alters patterns of hyporheic flow and influences solute mixing and nutrient transformations. Despite the abundance of research focused on hyporheic exchange and its ecological significance along river corridors, many studies neglect the details of sediment heterogeneity within compound bars, thereby overlooking various hydrologic and geochemical processes crucial to accurate characterization of fate and transport processes. The geophysical techniques applied in this study provide the missing piece needed to delineate the intricate sedimentary architecture of compound bars and to understand their influence on hyporheic zone processes in a minimally invasive manner. They facilitate spatial characterization of hydrologic and geochemical properties, which by proxy may reveal potential locations of reaction hotspots. When combined with sedimentological and hydrological measurements, the aggregate dataset provides a comprehensive, multifaceted understanding of hyporheic exchange and nutrient transformation processes. Incorporating such an integrated toolkit in the development of predictive models of many sites (e.g., Paula B. Matheus Carnevali et al., 2021; Dwivedi et al., 2018; Maavara et al., 2021) would help characterize spatial variability, and significantly improve predictive understandings of biogeochemical processes within the hyporheic zone. For example, airborne EMI for large-scale river systems (e.g., Minsley et al., 2012; Rey et al., 2019) could be used to upscale existing models and more broadly inform management strategies focused on maintaining water quality and ecological integrity. The demonstrated successful application of these geophysical methods at the TEMMS underscores their potential for broader applications, emphasizing their value in advancing river corridor science and in guiding conservation efforts (Crosato and Mosselman, 2020; Kasahara and Wondzell, 2003; Kasahara and Hill, 2007; Harvey and Bencala, 1993).

Cross-bar channel fills are essential to proper ecological function along river corridors not only because they impact hyporheic flow, but also due to their influence on subsurface nutrient transformations, which directly control downstream nutrient loading (Groffman et al., 2005; Harvey et al., 2012; Marzadri et al., 2012). As revealed through EMI surveys, cross-bar channel fills forge distinct physical and geochemical properties within the hyporheic zone (Figs. 2 and 3). From a physical standpoint, their low-*K* sediments act as baffles to groundwater flow, impeding solute transport and extending residence times. Consequently, they induce spatial variations in geochemical parameters that control the formation of hotspots and influence nutrient transformation processes such as denitrification (Figs. 8, 9 and Table 2). The hydrologic and geochemical importance of cross-bar channel fills has yet to be explored in other, more dynamic environments such as tidal rivers or within rivers under anthropogenic flow regimes imposed by dams. Regular stage fluctuations driven by such processes enhance hyporheic exchange within the riverbed and through the adjacent riparian floodplains along such rivers, altering the redox state of the groundwater system and inducing disparate zones of microbial activity based on the relative availability of complementary nutrients (e.g., Wallace et al., 2019, 2020). The presence of compound bars in such fluctuating systems would likely alter the already enhanced hyporheic exchange and drive the formation of hotspots within the riverbed. Cross-bar channel fills are dominant hydrobiogeochemical controls within riverbeds, and future research should focus on how they impact water quality in a wide variety of systems.

Combined groundwater monitoring and time-lapse ERI measurements within the compound bar provide critical insights into the dynamic interplay between sediment characteristics and subsurface hydraulic connectivity (Figs. 3 and 6). Fine-grained sediments within the cross-bar channel fill, particularly between PCB2 and PCB5, effectively act as a baffle to flow that alters hyporheic exchange patterns. The relative response of pore water elevations within the piezometer network during sampling coupled with time-lapse ERI data delineate preferential flow pathways that accentuate flow variability. These findings illuminate the profound effect of sedimentary architecture on hyporheic exchange and solute mixing, which ultimately influences nutrient cycling and the ecological functioning of the hyporheic zone (Zhou et al., 2014; Stonedahl et al., 2010). Specifically, the largest variations in electrical resistivity captured by ERI in the downstream portion of the bar correspond to regions with the most dynamic flow regimes (as indicated by the 3PE gradient network). The ability of cross-bar channel fills to modulate solute transport and hyporheic mixing becomes particularly pronounced during inundation events, during which hydraulic gradients shift and flow directions reverse. Capturing these temporal dynamics is vital for a nuanced understanding of solute residence times and for the accurate modeling of biogeochemical transformations along hyporheic flowpaths, with far-reaching implications for riverine ecosystem health.

Comparing piezometer locations to EMI data displayed in Fig. 6a, it would seem that the 3PE flow triangle representing PCB 4-6-7 should be impacted by cross-bar channel fill sediments acting as a baffle to flow; however, the data does not corroborate this with each of these piezometers behaving as if they have some degree of connectivity to the river and each other (Fig. 8). What must be considered is that EMI is responding to the bulk conductivity of a particular location that is driven by physical and chemical sediment characteristics as well as pore water chemistry. Because many variables are captured with EMI, results may not directly correlate with hydraulic connectivity. In this case, EMI results suggest that PCB4 and PCB5 are similar. It is more likely that PCB5 falls within the cross-bar channel fills and PCB4 is in a transitional area on the edge of the cross-bar channel fills where sediments transition to sands and gravels. This can be seen by comparing the hydraulic response of PCB4 that is more consistent with locations outside of cross-bar channel fills. It is possible that this piezometer is situated in a location consistent with the hotspots described by Wallace et al. (2019).

The detailed hydrogeochemical analysis across the compound bar reveals spatial heterogeneity in chemical environments that are intricately linked to the underlying sedimentary architecture. The formation of diverse geochemical zones likely supports a variety of ecological niches supporting distinct microbial communities sensitive to geochemical conditions (Sackett et al., 2019). Areas with high organic matter content, for instance, may support vigorous microbial activity, including aerobic and anaerobic respiration, denitrification, and other biogeochemical processes vital for nutrient cycling within aquatic ecosystems (Bertrand et al., 2015). This heterogeneity (see Figs. 8, 9 and Table 2) is essential for nutrient cycling within the hyporheic zone with specific areas acting as hotspots for microbial activity and biogeochemical processes and is vital for transforming nutrients like nitrogen, phosphorus, and carbon. This variability is closely tied to the underlying and multiscale sedimentary architecture (as shown conceptually in Fig. 1b), with larger scale features such as the cross-bar channel fill exhibiting distinct chemical signatures due to its unique smaller scale sediment composition and hydraulic properties (Fig. 2). Notably, temporal patterns in specific conductivity at PCB4 and PCB7 within and outside the cross-bar channel fills hint at complex interactions possibly linked to precipitation reactions in groundwater, underlining the importance of integrating microbiological insights for a more comprehensive understanding as hydrological and geochemical data alone do not provide adequate explanation. The interplay between these geochemical conditions and microbial life not only influences nutrient availability and the ecological health of the river system but also plays a critical role in the fate and transport of contaminants. Zones with specific redox conditions can facilitate the degradation or immobilization of pollutants, acting as natural filters that enhance water quality (Ford, 2005; Lawrence et al., 2013; Gandy et al., 2007; Dordio et al., 2008). Understanding the spatial extent and characteristics of these geochemically active zones is crucial for predicting the natural attenuation capacities of riverine environments and for designing effective river management and restoration strategies that leverage these natural processes to mitigate pollution. The geochemical zonation within the hyporheic zone is a pivotal factor influencing ecological processes and the overall health of riverine systems. By shaping the distribution and activity of microbial communities, controlling nutrient cycling, and affecting the fate of contaminants, these zones are integral to the functioning of aquatic ecosystems. Further research into the mechanisms driving geochemical variability and its ecological implications will be vital for preserving and enhancing the ecological integrity of riverine environments.

Mineralogy across the bar also provides insights into depositional history, sediment provenance, and processes at play within the bar post-deposition. A prime example is the presence of glauconite within cross-bar channel fills but not within the rest of the bar. Glauconite is likely sourced from carbonates of the Dayton and Waco formations that are noted for having high concentrations of glauconite (Brett et al., 2012; Waid and Brett, 2019). Glauconite was presumably inherited via sediment transport as it is known to form authigenically in saltwater but not freshwater (Nelsen et al., 1994). Glauconite is preserved within the cross-bar channel fill sediments potentially due to favorable geochemistry (i.e., reducing to preserve ferrous iron in structure). The absence of glauconite outside of the cross-bar channel fills may be a result of slow weathering resulting in conversion of glauconite to minerals such as kaolinite and iron oxyhydroxides (Skiba et al., 2014).

## 6. Conclusions and future directions

The comprehensive analyses presented in this study knit together strands of evidence from hydrology, geophysics, geochemistry, and geomorphology to build a multifaceted understanding of hyporheic zone functioning within compound bars. By combining piezometer data, geophysical imaging, sediment characterization, and geochemical profiling, this study provides fundamental understanding on the dynamic interactions that govern riverine hydrogeochemistry, depicting a dynamic system influenced by sedimentary heterogeneity, hydrological connectivity, and likely microbial activity. These interactions highlight the intricate balance between physical processes and biogeochemical reactions that determine the hyporheic zone’s role within the larger ecological context of the river system underlining the need for understanding multiscale morphology and sediment heterogeneity for maintaining the quality and sustainability of the water resource and ensuring the ecological health of the river system. The implications of this research are broad, informing both scientific understanding and practical management of riverine environments.

Though the underlying sedimentary architecture of compound bars ultimately controls the spatial distribution of reaction hot spots in the adjacent hyporheic zone, they vary temporally in response to hydrologic perturbations (i.e., storms). Further, as the magnitude of hydrologic perturbation increases and infiltrating surface water delivers more nutrients, the geochemical importance of features such as cross-bar channel fills will likely magnify. To capture the geochemical dynamics during such episodic flow events, continuous in-situ redox monitoring could be implemented. Redox potential is often related to the primary concentrations of redox-active species (e.g., NO_3_^−^), and is thought to be indicative of various microbial processes (Ferris and Szponar, 2021). It has also been used to describe the potential for degradation of anthropogenic contaminants in aquifers (Lensing et al., 1994; McMahon and Chapelle, 2008). Wallace and Soltanian (2021b) applied continuous 3D monitoring of redox potential in the TEMMS riparian floodplain to assess the geochemical response to sediment heterogeneity and water table fluctuations. They observed that redox potential increased at some locations following river water infiltration during a storm, while persistent zones of high potential endured along preferential high-*K* flow paths. Their study highlights that redox potential fluctuations were most significant at or near the transition between low- and high-*K* sediments. It is likely that such fluctuations could be observed between cross-bar channel fills and their surrounding within compound bar deposits. Additionally, dissolved organic matter chemistry is becoming increasingly recognized as having strong influences over biogeochemical reaction rates within the hyporheic zone (Graham et al., 2017; Song et al., 2020). Mixing of DOC with varying thermodynamic favorabilities from river water during stage fluctuations has been shown to initiate spikes in microbial activity (Stegen et al., 2018). Exploring the organic matter present in the river and groundwater could provide a more thorough understanding of the reaction processes taking place within compound bars, such as denitrification and sulfate reduction based on the low DO observed. Examining sulfate reduction in the cross-bar channel fill samples would provide more insight on this. Further, examining the microbes present and their metabolic activities through metagenomic and metatranscriptomic analysis of these zones would allow us to confirm the activity and presence of the organisms driving these reactions.

**Fig. 10.**
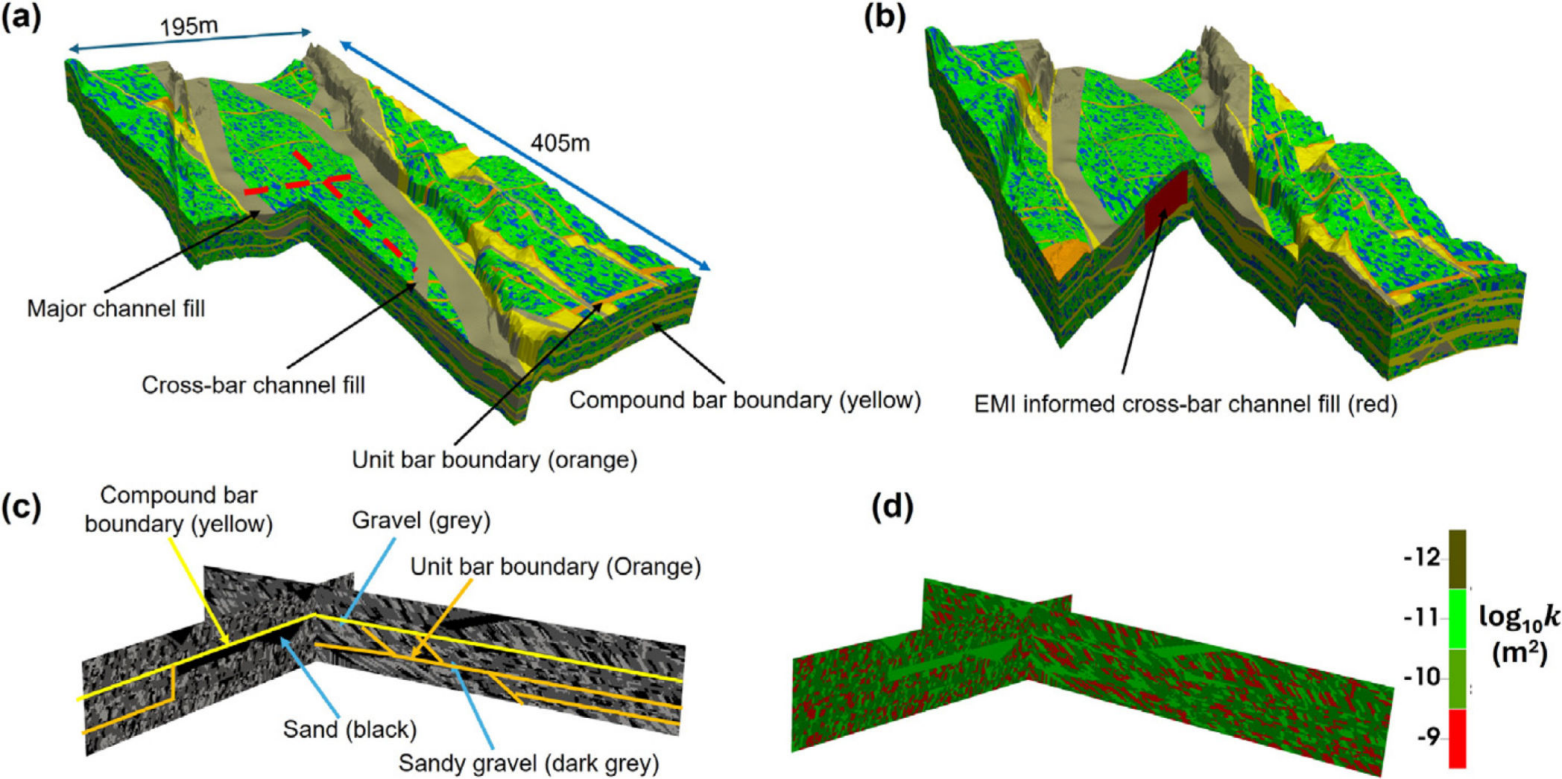
(a) An example of GEOSIM realization including compound bar, river channels, and floodplain at TEMMS. SfM DEM data combined with riverbed topography data collected using an acoustic doppler current profiler (ADCP) used to generate surface topography. Red lines show locations of cross sections in (c) and (d). (b) GEOSIM realization modified using observed EMI data to add the cross-bar channel fill shown in Fig. 3. (c) and (d) Cross sections of small scale cross strata of sand, sandy gravel, and gravel and the corresponding permeability field populated using observed log-normal distribution for each cross strata.

The results of this work deepens our understanding on the impacts of geomorphology and sedimentary architecture heterogeneity, especially the role of cross-bar channel fills, on flow and solute transport processes in the hyporheic zone. To provide more insight into overall hyporheic flow dynamics and reactive transport processes and make this work transformative with applications to other compound bars, developing high-resolution and 3D numerical laboratory models that incorporate 3D geomorphology and sedimentary architecture heterogeneity across scales are needed. To do this, one can use advanced geocelluar modeling tools such as geometric-based simulation methods (Ramanathan et al., 2010; Guin et al., 2010). Such approaches generate the most important and relevant features of multiscale fluvial architecture (Gershenzon et al., 2014, 2015; Ershadnia et al., 2021). Fig. 10 presents one realization from such simulation produced using GEOSIM (Ramanathan et al., 2010; Guin et al., 2010), which is a digital model for the hierarchical and multi-scale fluvial architecture. GEOSIM is well-suited to creating hierarchical architecture in which a facies type at one scale forms the bounding surfaces for an assemblage of smaller facies types at the next lower scale, each of those a bounding surface for facies types still smaller in scale. GEOSIM uses geometric polygons using piecewise-planar elements to simulate different facies across and at different scales (illustrated in Ramanathan et al. (2010), Fig. 7 for one sediment type: a unit bar deposit). Each type of facies has an archetypal geometry but the length and angle parameters defining the plane equations for each occurrence are drawn randomly from distribution functions as found in nature (mostly Erlangian), giving natural variation around the archetypal shape. The geometric shapes of larger scale features form the bounding surfaces of assemblages of the features defined at the next lower scale. As space is filled at each successively lower scale, parts of one facies take precedence over parts of others based on rules related to the sequential order of sediment erosion and deposition (Ramanathan et al., 2010). The model is continuously defined in space and is discretized at whatever resolution is required. For example, the grid spacing can match that used in a flow model. The code assigns a hierarchy of integer values indicating facies types at different scales (e.g., smaller scale cross strata to larger scale features such as cross-bar channels and major channel fills). The topography and riverbed geometry from SfM and other data types such as riverbed topography (e.g., from acoustic doppler current profiler (ADCP)) could be integrated. Fig. 10 shows an example integrated lidar drone data with hierarchical discrete stratal architecture generated by GEOSIM.

It is important to incorporate a three-dimensional sedimentary architecture model generated by GEOSIM into flow and reactive transport models to enhance understanding of the hyporheic exchange and the resulting geochemical dynamics. Modeling studies have investigated the influence of sediment architecture heterogeneity on hyporheic exchange processes. Results from these studies demonstrate that sediment heterogeneity significantly affects the flow path; thus controls the location and magnitude of subsurface biogeochemical reactions (e.g., transformation of nitrate) (Sawyer, 2015; Pescimoro et al., 2019; Wallace et al., 2021; Laube et al., 2022). However, these studies either used two-dimensional models or only included a simple, single-scale representation of sediment heterogeneity. Two-dimensional models are not able to capture the full complexity of sediment heterogeneity and 3D geometries as well as connectivity of high-*K* sediments across scales as the digital models such as GEOSIM able to produce. Perhaps the most important question that could only be answered by using GEOSIM type models is which scale of sediment heterogeneity is most relevant to understanding and predicting hyporheic zone processes. Another advantage is the ability to constrain GEOSIM type models using geophysical measurements. For instance, in Fig. 10b the GEOSIM model was modified to include the cross-bar channel fill observed in EMI. The authors are currently incorporating GEOSIM models of the TEMMS compound bar into fully coupled flow and reactive transport simulations.

The ecological significance of our findings, particularly regarding geochemical zonation within the hyporheic zone, suggests potential implications for river ecosystem health and function that merits further investigation. This study underscores how sedimentary architecture intricately shapes geochemical gradients, which in turn influence the river’s resilience to nutrient loading and pollution. Such zonation creates distinct niches that support a diverse array of microbial communities, pivotal in mediating biogeochemical cycles. These microbial hotspots are essential for transforming and mitigating excess nutrients and contaminants, thereby acting as a natural filtration system that enhances water quality. Moreover, the spatial variability in chemical and physical properties within compound bars contributes to the overall biodiversity of river ecosystems by providing varied habitats for microbial communities. Future study should examine the relative contributions internal and external to compound bars for transforming and mitigating excess nutrients and contaminants, to determine the ecological role that compound bars play. This study provides perspective for aspects that should be investigated for components of the river system external to the compound bar, which could provide a common framework for assessing relative contributions to river ecosystem health and function. In the face of anthropogenic impacts and climate change, it is important to understand the role of the hyporheic zone’s dynamic geochemical zonation in sustaining river health.

## Data availability

A link to the data has been added in the text. We will publish it with acceptance of this paper.

## Supplementary Material

Supplementary Material

## Figures and Tables

**Table 1:** Summary of topographic data collections and key changes.

Date	Stage at Miamitown (m)	Peak stage between flights (m)	Notes	Geomorphic changes since last observation

2019-09-04	0.35	–	Baseflow	
2021-09-14	0.25	7.2	Baseflow	Migration of cross-bar channel and its constraining unit bar by 40 m
2022-07-11	0.96	5.7	Mid-channel bar 90% submerged	Further migration of cross-bar channel and its constraining unit bar by 15 m
2022-07-15	0.61	0.96	Low flood stage	
2022-07-21	1.1	2.2	Mid-channel bar 100% submerged	
2022-08-11	0.43	1.1	Low flood stage	Removal of woody debris from top of unit bar; changes to minor bedforms
2022-08-19	0.33	0.43	Baseflow	
2023-06-06	0.37	6.3	Baseflow	Little migration of cross-bar channel and 10 m movement of constraining unit bar front; emergence of new following unit bar

**Table 2:** Physical and chemical properties of water samples collected on August 22, 2022. The n.a. indicates that the data is not available due to non-detectable concentrations.

Measurements	RW	PCB1	PCB2	PCB3	PCB4	PCB5	PCB6	PCB7	PCB8

Temp (C)	24.39	26.00	24.89	26.39	25.28	24.00	25.83	21.28	22.28
Conductivity (μS/cm)	820	725	1494	719	914	2341	665	868	787
pH	8.7	7.7	7.1	7.8	7.3	7.7	7.5	7.5	7.6
DO (% saturation)	101.5	3.8	4.0	4.0	9.8	5.0	5.9	5.0	5.2
DOC (mg/L)	2.45	2.43	39.11	2.43	6.68	60.93	3.65	1.99	2.41
**Anaerobic electron acceptors (mg/L)**									
N as NO_3_^−^	24.306	0.016	0.617	n.a.	n.a.	42.305	n.a.	0.350	0.026
N as NO_2_^−^	0.268	n.a.	4.648	1.8306	0.2845	2.2988	2.2844	2.4798	3.4989
SO_4_^2−^	122.65	117.53	5.37	99.00	4.49	5.19	62.46	138.08	144.69
**Electron donors (mg/L)**									
Fe^2+^	0.02	0.29	11.5	0.13	1.79	0.55	0.27	1.1	0.87
**Other important chemical parameters (mg/L)**									
NH_4_^+^	n.a.	1.42	48.67	2.01	24.07	87.59	4.19	1.86	1.81
