# Supplementary material for: Uncovering the hidden world of riverbed sediments: The role of sediment heterogeneity and cross-bar channel fills in the hydrogeochemical dynamics of the hyporheic zone
Source: J Hydrol (Amst). Author manuscript; Available in PMC 2025 Nov 1. (PMC11960729; doi:10.1016/j.jhydrol.2024.132062)
Supplement: Supplementary Material [file NIHMS2038707-supplement-Supplementary_Material.docx]

**Supporting Information for “****Uncovering the hidden world of riverbed sediments: The role of sediment heterogeneity and cross-bar channel fills in hydrogeochemical dynamics of the**

**hyporheic zone”**

Jeffery Tyler McGarr^a*^, Pei Li^a^, Robert G. Ford^b^, Teagan Kleman^a^, Colton Fields^a^, Julie Hobbs^a^, Lydia Lupton^a^, Emma Poston^a^, Thomas Marsh^a^, Leah Trutschel^c^, Ken M. Fritz^b^, Annette Rowe^c^, Corey D. Wallace^1^, Dylan Ward^a^, Daniel M. Sturmer^a^, Craig Dietsch^a^, Margaret Naber^a^, Bob K. Lien^b^, Mohamad Reza Soltanian^a,d**^

^a^Department of Geosciences, University of Cincinnati, Cincinnati, OH, USA

^b^Office of Research and Development, U.S. Environmental Protection Agency, Cincinnati, OH, USA

^c^Department of Biological Sciences, University of Cincinnati, Cincinnati, OH, USA

^d^RSI EnTech, Grand Junction, CO, USA

^e^Department of Environmental Engineering, University of Cincinnati, Cincinnati, OH, USA

^*^Corresponding author

^**^Corresponding author

Email address: mcgarrjt@mail.uc.edu (Jeffery T. McGarr), soltanma@uc.edu (Mohamad Reza Soltanian)

**Contents of this file**

Figures S1 to S8

**Introduction:** The supporting information includes a gif of historical site imagery, the inversion errors for EMI, and various hydrological plots. For ERI inversion error plots and meshes, the reader is directed to McGarr et al. (2021).


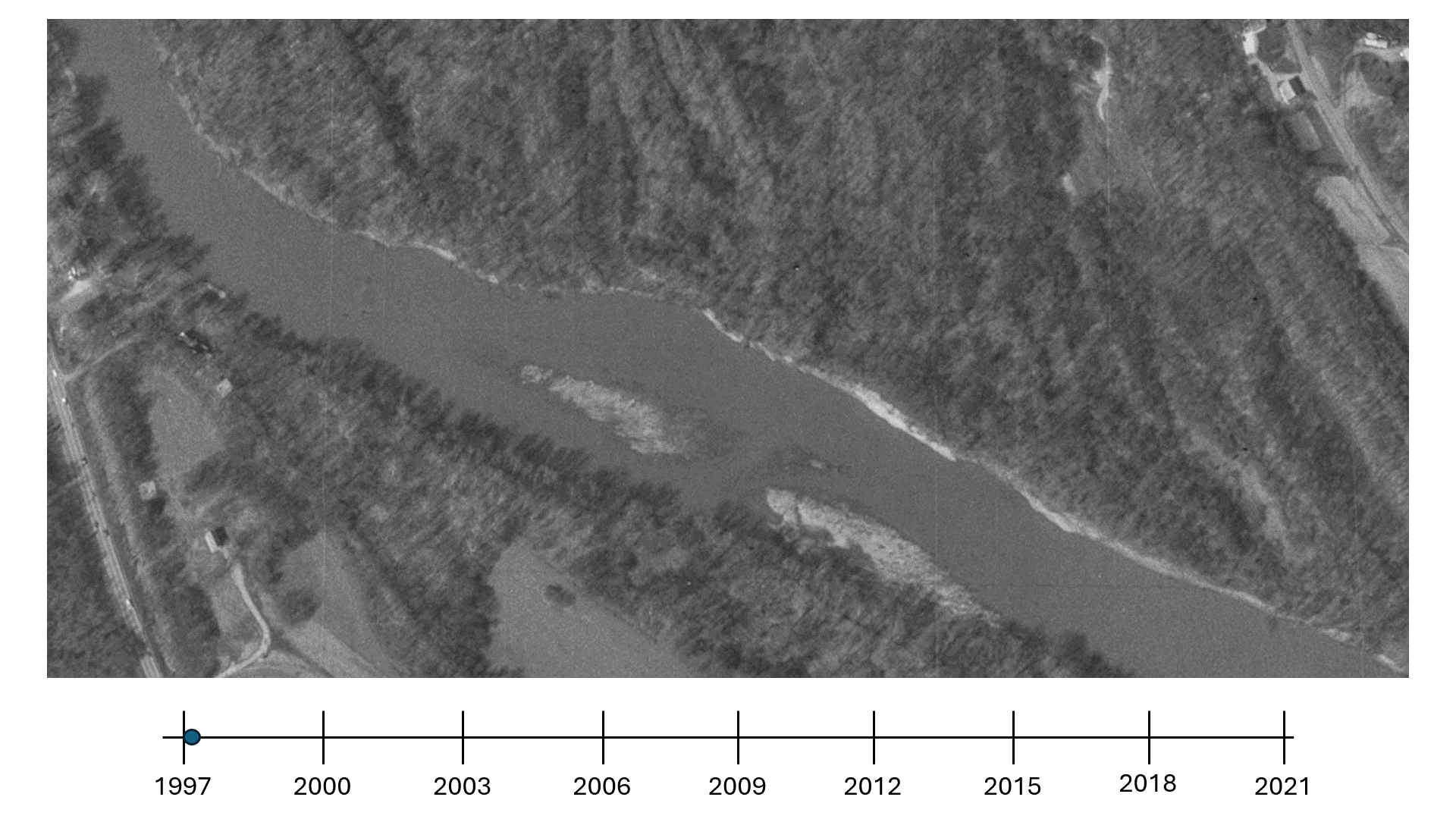


Figure S1: Aerial imagery of the compound bar in this study from March 1997 to August 2019 acquired on Google Earth.


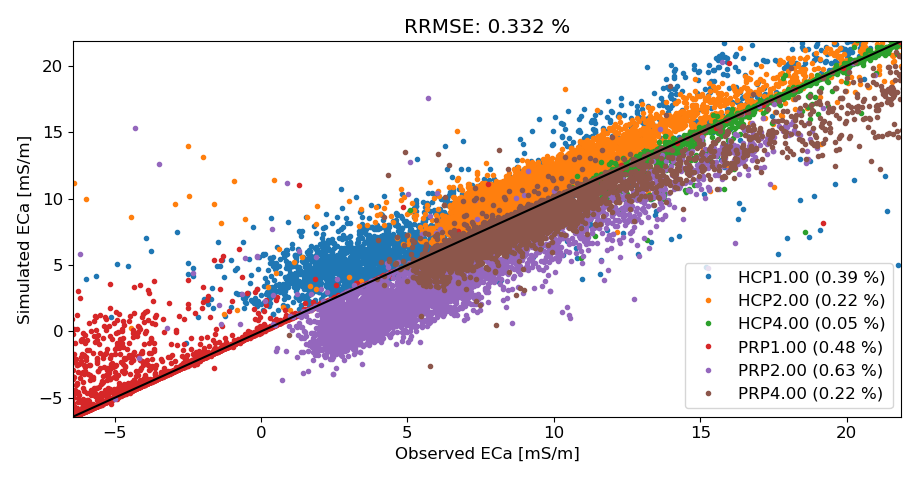


Figure S2: Relative root mean square error (RRMSE) of inverted EMI data overall and for each sensor as calculated by EMagPy. The overall RRMSE is 0.332. RRMSE for each sensor is shown in the chart with naming conventions being sensor alignment followed by transmitter/receiver spacing in meters. HCP is the horizontal coplanar array. PRP is the perpendicular array (Ex. PRP-1m is the perpendicular transmitter/receiver array with a spacing of 1 m).


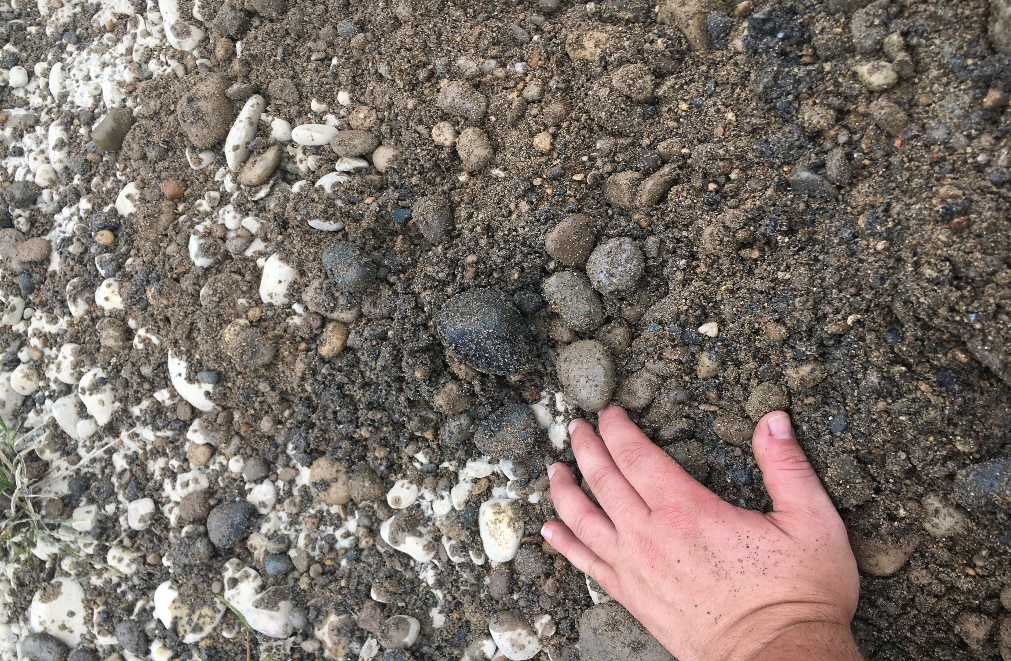


Figure S3: Picture of the sands and gravels that make up the majority of the bar. Referred to as non-XB in the text.


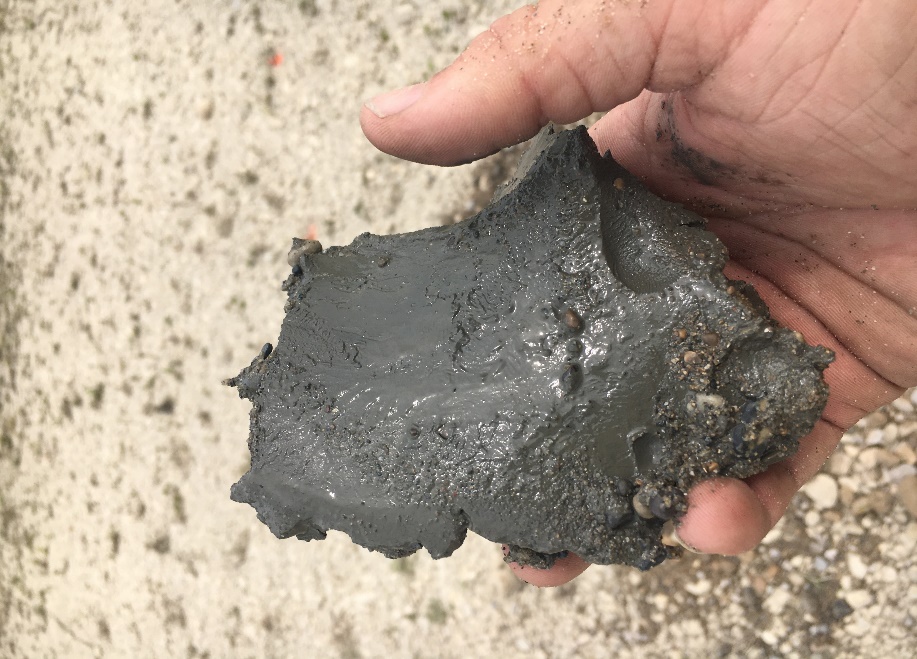


Figure S4: Picture of the fine grained cross-bar channel fill sediments. Referred to as XB in the text.


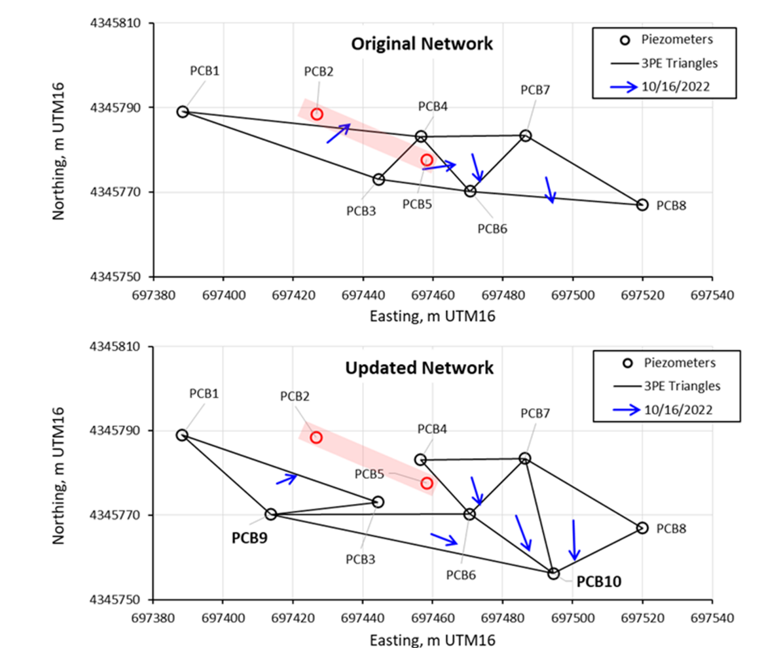


Figure S5: Using 3PE to identify “problems” at the TEMMS compound bar (CB). Original piezometer (“PCB”) network was determined to target locations that showed geologic discontinuities (see top plot). Piezometers PCB2 and PCB5 coincided with locations previously identified within a cross-bar channel with differences in sediment characteristics.

Lithologic information along with subsequent hydrologic data indicated that piezometers PCB2 and PCB5 should not be included in 3PE estimates of hydraulic gradients (see red circles/rectangle). Based on this analysis, additional piezometers PCB9 and PCB10 were installed in locations that seemed logical for groundwater flow based on CB hydrostratigraphy

Re-examination of hydraulic gradient data with new piezometer network indicates that there is a barrier to groundwater flow across a zone bounded by piezometers PCB2 and PCB5, consistent with hydrostratigraphy data. For orientation of the piezometer network on the bar please reference Figure 1 in the main text. River flow direction is generally from left to right on these plots.


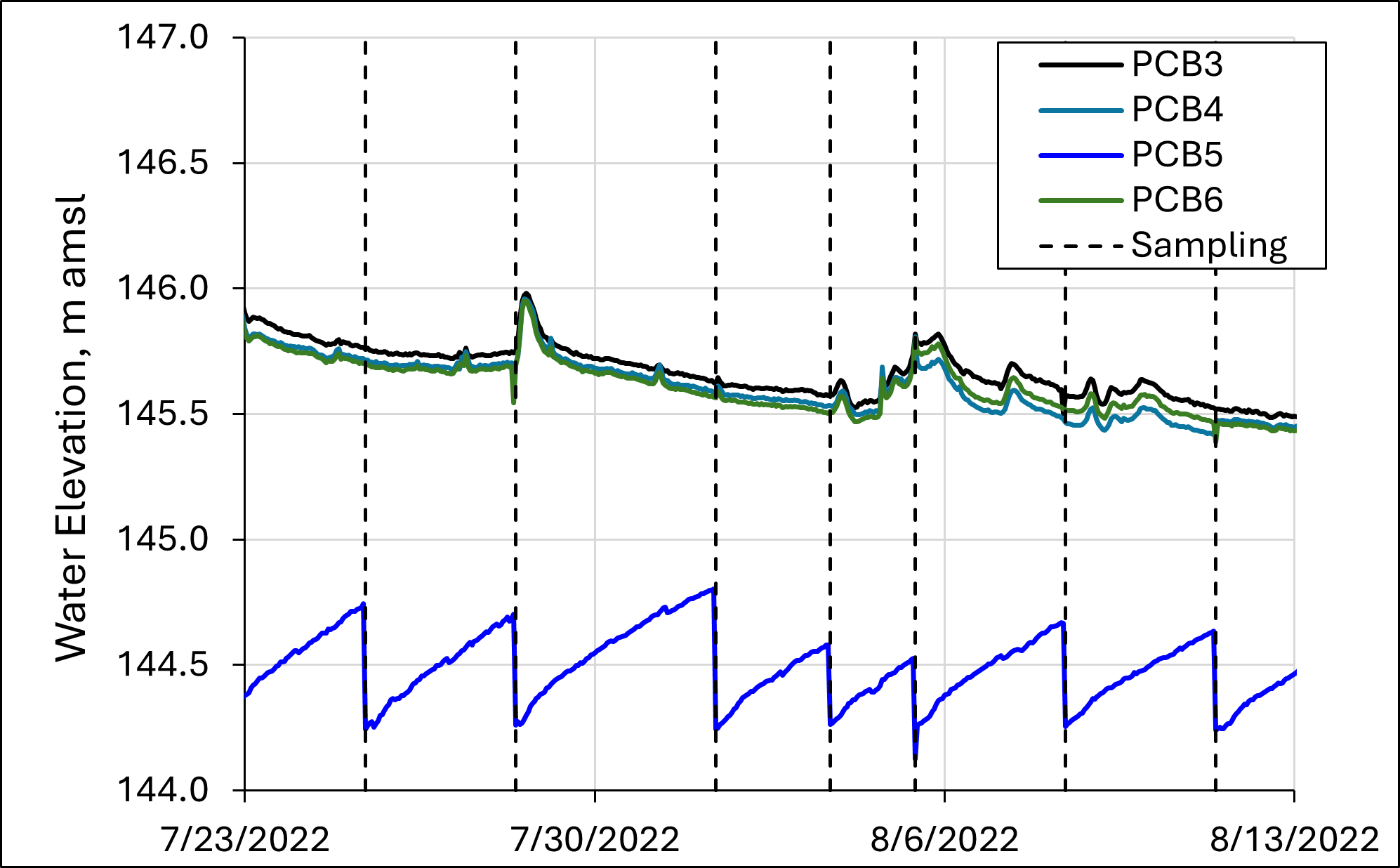


Figure S6: Patterns of hydraulic head at PCB3, PCB4, PCB5, PCB6 during sampling. PCB5 displays saw-tooth pattern in groundwater elevation initiated by sharp draw-down during sampling. A few instances of draw-down in PCB3 and PCB6, but less significant and groundwater elevation recovers quickly. The draw-down and slow recovery in PCB5 indicates that the CB sediments at this location do not transmit groundwater as readily as the other locations. This behavior is consistent with observation of sediment characteristics (e.g., grain size); see McGarr et al. 2021 and sediment characteristics within this work. 3PE triangle PCB3-PCB4-PCB6 was determined to be inappropriate due to discontinuous geology


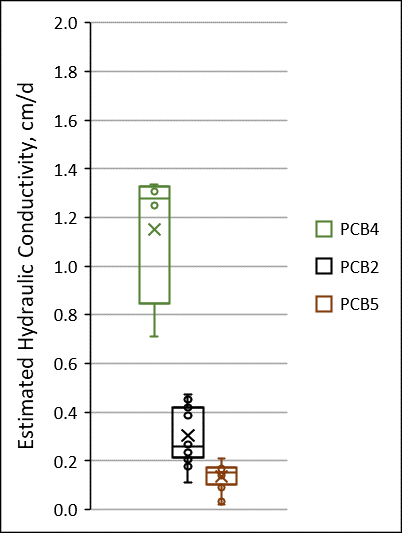


Figure S7: Plot of estimated hydraulic conductivity (*K*) (cm/d) of PCB2, PCB4, and PCB5. Estimated hydraulic conductivity at PCB2, PCB4, and PCB5 based on record of drawdown and recovery following sampling (i.e., similar to a rising-head slug test). This should be considered a crude estimate since the rate of data logging and period of recovery monitoring was not optimized as one would do for a conventional slug test. This was not feasible for the other locations since there was no significant drawdown relative to the hourly transducer logging rate.


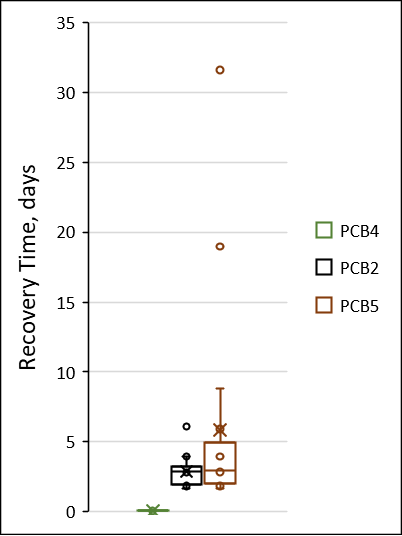


Figure S8: Plot of recovery times (time to equilibrium post-sampling event) of PCB2, PCB4, and PCB5. For PCB2 and PCB5, the sequence of sampling occurred before the piezometer could fully recover to a static water level; longest recovery times for both were closest to the period needed to fully recover (PCB2 6 days, *K_h_*,est 0.2 cm/d; PCB5 32 days, *K_h_,*est 0.02 cm/d). This was not feasible for the other locations since there was no significant drawdown relative to the hourly transducer logging rate.


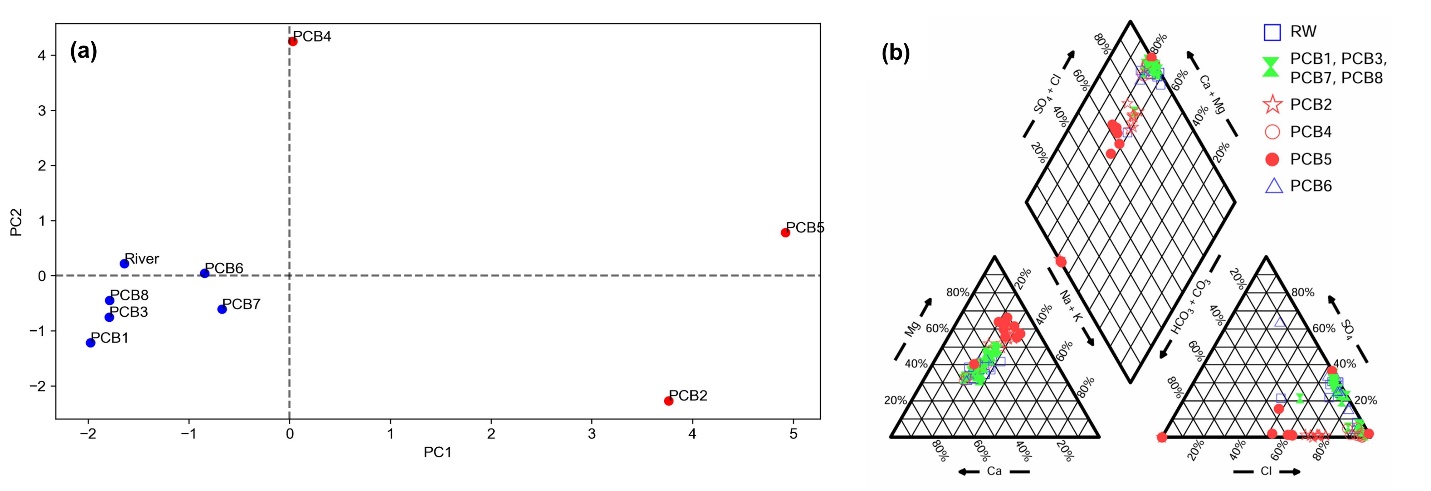


Figure S9: (a) Principal Component Analysis (PCA) of the geochemical data with red circles showing data from PCB2, PCB4, and PCB5 and blue circles showing data from the remaining piezometers and the river. PCB2 and PCB5 are located within the cross-bar channel fill. PCB4 is located in a transitional zone between the cross-bar channel fill and the rest of the bar that is more hydraulically connected to the river. (b) Piper diagram with select piezometers and river water collected July 25, 2022 through August 22, 2022 across eight sampling events.

**References**

McGarr, J. T., Wallace, C. D., Ntarlagiannis, D., Sturmer, D. M., & Soltanian, M. R. (2021). Geophysical mapping of hyporheic processes controlled by sedimentary architecture within compound bar deposits. Hydrological Processes, 35(9), e14358.
